# Air pollution exposure is linked with methylation of immunoregulatory genes, altered immune cell profiles, and increased blood pressure in children

**DOI:** 10.1038/s41598-021-83577-3

**Published:** 2021-02-18

**Authors:** Mary Prunicki, Nicholas Cauwenberghs, Justin Lee, Xiaoying Zhou, Hesam Movassagh, Elizabeth Noth, Fred Lurmann, S. Katharine Hammond, John R. Balmes, Manisha Desai, Joseph C. Wu, Kari C. Nadeau

**Affiliations:** 1 Sean N. Parker Center for Allergy and Asthma Research at Stanford University, Stanford, CA 94305 USA; 2 Department of Cardiovascular Sciences, University of Leuven, Leuven, Belgium; 3 Department of Medicine, Stanford University, Stanford, CA 94305 USA; 4 Quantitative Sciences Unit, Stanford University, Stanford, CA 94305 USA; 5 School of Public Health, University of California, Berkeley, Berkeley, CA 94720 USA; 6 Sonoma Technology, Inc., Petaluma, CA 94954 USA; 7 Department of Medicine, University of California, San Francisco, CA 94143 USA; 8 Stanford Cardiovascular Institute, Stanford University, Stanford, CA 94305 USA; 9 Division of Pulmonary and Critical Care Medicine, Department of Medicine, Sean N. Parker Center for Allergy and Asthma Research at Stanford University, Stanford University, Stanford University School of Medicine, 269 Campus Drive, CCSR 3215, MC 5366, Stanford, CA 94305-5101 USA

**Keywords:** Immunology, Environmental sciences, Natural hazards, Cardiology, Medical research, Risk factors

## Abstract

Ambient air pollution exposure is associated with cardiovascular dysregulation and immune system alterations, yet no study has investigated both simultaneously in children. Understanding the multifaceted impacts may provide early clues for clinical intervention prior to actual disease presentation. We therefore determined the associations between exposure to multiple air pollutants and both immunological outcomes (methylation and protein expression of immune cell types associated with immune regulation) and cardiovascular outcomes (blood pressure) in a cohort of school-aged children (6–8 years; n = 221) living in a city with known elevated pollution levels. Exposure to fine particular matter (PM_2.5_), carbon monoxide (CO), and ozone (O_3_) was linked to altered methylation of most CpG sites for genes Foxp3, IL-4, IL-10 and IFN-g, all involved in immune regulation (e.g. higher PM_2.5_ exposure 1 month prior to the study visit was independently associated with methylation of the IL-4 CpG24 site (est = 0.16; *P* = 0.0095). Also, immune T helper cell types (Th1, Th2 and Th17) were associated with short-term exposure to PM_2.5_, O_3_ and CO (e.g. Th1 cells associated with PM_2.5_ at 30 days: est = − 0.34, P < 0.0001). Both B cells (est = − 0.19) and CD4+ cells (est = 0.16) were associated with 1 day NO2 exposure (P ≤ 0.031), whereas CD4+ and CD8+ cells were associated with chronic exposure to PAH_456_, NOx and/or NO_2_ (*P* ≤ 0.038 for all). Finally, diastolic BP (DBP) was inversely associated with long-term exposures to both CO and PAH_456_, and both systolic and pulse pressure were associated with short-term NO_2_ and chronic NOx exposure. Our findings demonstrate links between air pollution exposure and methylation of immunoregulatory genes, immune cell profiles and blood pressure, suggesting that even at a young age, the immune and cardiovascular systems are negatively impacted by exposure to air pollution.

## Introduction

The World Health Organization (WHO) estimates that 93% of the world’s children under 15 years—1.8 billion children—breathe air that puts their health and development at risk^1^ and more than 25% of all children in developed countries develop disorders linked with immune system dysfunction from air pollution exposure^2,3^. After respiratory disorders, cardiometabolic disorders are the most important diseases attributed to exposure to ambient air pollution^4^. Despite these links between air pollution and disease, few studies have investigated the effects of ambient air pollution on both the immune and cardiovascular system simultaneously, especially in children^5^.

Indeed, research on the impact of pollution on children’s immune status and cardiovascular health is limited in comparison to the adult literature. A study of 366 school-aged children (9 to 11 years) from Central Europe found that air pollution increased B cells, CD4+ and CD8+ T cells, and natural killer cells with increasing exposure to particulate matter (PM)^6^. While it is known that PM_2.5_ increases blood pressure (BP) in adults^7^, there have been fewer studies in children, with some, but not all, finding an association between PM and BP: Children exposed to elevated concentrations of ultrafine to coarse PM^8^ or PM_2.5_ and NO_2_ had increased systolic and diastolic blood pressures^9^. In addition, PM was associated with hypertension in children and adolescents in China^10^. Finally, higher PM_2.5_ exposure during pregnancy was associated with elevated blood pressure in children ages 3 to 9 years^11^.

Here we simultaneously investigate the effects of ambient air pollutants on both the immune status and cardiovascular health in children. Our hypotheses are that pollution exposure will be linked with: (1) Methylation levels of important immunoregulatory genes (Foxp3, IL-4, IL-10 and IFNγ), (2) Immune protein expression as defined by immune cell profiling, and (3) Systolic and diastolic BP and pulse pressure. We therefore determined the associations between prior exposure to a large set of air pollutants and both immunological outcomes (methylation and protein expression of immune cell types associated with immune regulation) and cardiovascular outcomes (blood pressure levels) in a cohort of school-aged children (6–8 years of age) living in Fresno, California, a city with known elevated pollution levels. Importantly, we use the novel technology of mass cytometry by time of flight (CyTOF) to more sensitively measure 30–40 cell markers simultaneously, providing a more in-depth analysis of pollution exposure impacts than previously possible.

## Materials and methods

### Guidelines and ethical statement

All participants in this study gave written informed consent for the protocol that was approved by Stanford University’s Institutional Review Board (Ethics approval number 28263). Informed consent of the children in this study was obtained from their parents or legally authorized representatives or guardians. This study was conducted in accordance with the Declaration of Helsinki that covers informed consent, privacy and confidentiality, ethical considerations, and assessment of any risks. This study followed all relevant guidelines of clinical research including clinical value, scientific validity, fair subject selection, favorable risk–benefit ratio, independent review, informed consent, and respect for potential and enrolled participants.

### Study design

From 2015 to 2016, eligible children (6 to 8 years) attending school in Fresno, California, visited our University of California San Francisco-Fresno clinical site, where we obtained a detailed health and demographics questionnaire, BPs, and blood samples. Over the 1-year study period, we measured air pollutants using a combination of continuous daily pollutant concentrations measured at central air monitoring stations in Fresno, daily concentrations from periodic spatial sampling, and meteorological and geophysical data. Average air pollution exposures were estimated for 1 day, 1 week and 1, 3, 6 and 12 months prior to each participant visit. Using high-dimensional mass cytometry (CyTOF), we determined immune cell markers from unstimulated participant peripheral blood mononuclear cells (PBMCs). Details of pollution and cell measures are described below.

### Study population recruitment and inclusion

We recruited a cohort of school-aged children (≥ 6 years) from the Fresno Unified school district, encompassing 5 schools with our enrollment log having an even distribution from each school*.* Recruitment was broad and intended to include all children who met the inclusion criteria. Recruitment posters were distributed throughout all schools and flyers were distributed to all students with their first day of school materials. Children were also recruited through school nurses, advertisements, physicians’ offices, and local media. Recruitment procedures were those previously described in the Fresno Asthmatic Children’s Environment Study (FACES)^12^. We determined eligibility criteria, which included children 6 to 8 years old and living within a 20-km radius of the CARB air quality monitoring sites in Fresno for at least 3 months. All children in the study were English speakers; parents were required to be fluent in either English or Spanish. Families who had plans to move from the area within the next year were excluded, as were children who did not spend at least 4 nights a week in one residence. Children were also excluded if they had taken any type of oral immuno-suppressants within 5 days of the blood draw, had a history of allergen immunotherapy within 1 year of the clinical visit, had a chronic disease other than allergies or asthma, or had an acute infection. Children with asthma were defined by the participant’s parent report of a physician prior diagnosis of asthma. Secondhand smoke exposure was defined as exposure to cigarette smoke from other household members. During the study period, we enrolled 221 eligible children in total.

### Air pollution exposure estimation and analysis

According to published methods^13^ continuous daily pollutant concentrations from four air quality monitoring stations located within the Fresno city limits, periodic spatial sampling, and meteorological and geophysical data were used to assign exposures to the following pollutants as described in the statistical analyses section and elsewhere: 5-, and 6-ringed polycyclic aromatic hydrocarbons (PAH_456_), PM_2.5_, ozone (O_3_), carbon monoxide (CO), elemental carbon, nitrogen dioxide (NO_2_), and nitrogen oxides (NO_x_). Hourly concentrations of particle-bound PAHs were measured at each monitoring station with the PAS2000 (EcoChem Analytics, League City, TX). Spatial–temporal models that used the air quality data along with meteorological and land-use data, were used to estimate concentrations at each participant’s residence^14^. AAP exposures were estimated 1 day prior to each participant’s clinical visit and also averaged over 1 week and 1, 3, 6 and 12 months prior to each participant visit. Individual exposure estimates were calculated based on the distance of the monitoring station to the participant’s home using published techniques^13,15^. The air pollution data were subject to rigorous and frequent checks for quality assurance, including range checks, comparison of values at nearby monitoring sites, and consistency with historical temporal and/or diurnal patterns for each pollutant. Supplemental Fig. S1 shows the longitudinal mean exposure over time PM_2.5_, O_3_, PAH_456_, NO_2_, elemental Carbon, NOx, and CO in five different stations at Fresno. Participant visits were scheduled on different days throughout the year, providing us with a wide ambient air pollution distribution across individuals from 1 day through 12 months prior to the study visit for the seven different pollutants.

Random effects regression was used to develop spatial–temporal models of daily concentrations for PAH_456_, EC, and NO_2_ using data from 2002 to 2015 field sampling campaigns in Fresno and Clovis^16^. Sampling location and date were treated as random effects to simultaneously capture the temporal and spatial components. Covariates considered for each exposure model include the continuously measured daily pollutant concentrations at fixed sites, relative humidity, temperature, wind speed, atmospheric stability, distance to nearest freeway. The models for PAH_456_, EC, and NO_2_ are able to explain 53%, 95%, and 99% of the observed temporal variance, and 74%, 88%, and 74% of the observed spatial variance, respectively, during the periodic spatially intensive sampling campaigns. Daily concentrations of air pollutants reflect 24 h of exposure, noon-to-noon, ending on the date of the participant’s visit to the study (“test date”).

### Study visit procedures

At our University of California San Francisco-Fresno clinical site, a detailed health and demographics questionnaire was completed by the accompanying parent, and BPs were measured by a nurse prior to blood draw according to the CDC National Health and Nutrition Examination Survey protocol^17^. Non-fasting blood samples were collected using validated techniques. Human PBMCs and plasma were extracted from blood via Ficoll procedure and stored in liquid nitrogen as per published techniques^18^.

### Blood processing

Blood samples were shipped overnight to Stanford University and peripheral blood mononuclear cells (PBMCs) and plasma were extracted from blood samples via Ficoll procedure; PBMCs were stored in liquid nitrogen and plasma stored at − 80 °C, per published techniques^18^.

### Methylation measurement

Based on previous published studies^19–21^, we chose key sites of important immunoregulatory genes that modulate regulatory T cell (Treg) response and overall immune tolerance: (1) 2 CpG sites in the promoter regions of the Foxp3 gene (human genome (hg38) Chr X 49264956 and 49264916), (2) 2 CpG sites in the promoter region and 4 CpG sites in the intron 2 region of the IL-4 gene (Chr5:132673907, 132673938; 132675133, 132675115, 132675095, 132675242), (3) 4 CpG sites in the intron 4 region of the IL-10 gene (Chr1206769266, 206769234, 206769230, 206769214) and (4) 3 CpG sites in the promoter region of the IFNγ gene (Chr12: 68160040, 68159930, 68159798). Samples were bisulfite treated, PCR amplified, pyrosequenced and percent methylation was obtained by EpigenDx (Hopkinton, MA, USA). Acceptable assay precision was where 90% of the samples had an average methylation value of ± 2.5 Standard Deviation (SD) except for the limit of quantitation, where it should not exceed ± 2 SD. We also used a coefficient of variation of 5% as a cutoff. Supplemental Table S1 shows the mean percent methylation and standard deviation for all subjects for each gene/CpG site.

### High-dimensional immunophenotyping measurement

Cell type measurement on unstimulated PBMCs was performed using a high-throughput, time-of-flight mass cytometry approach, CyTOF, to characterize immune cell subsets at a single-cell level. CyTOF is based on inductively coupled plasma mass spectrometry and time of flight mass spectrometry used to identify properties of cells using heavy metal ion tags rather than fluorochromes. See Supplemental Table S2 for the CyTOF panel. Premade MAXPAR antibodies against the immune cell markers of interest were purchased through Fluidigm Corporation (South San Francisco, CA, USA). PBMCs were stained for surface markers using validated, published methods^22–24^. Briefly, cryopreserved PBMCs were thawed and incubated in RPMI 1640 media (ThermoFisher) supplemented with 10% FBS at 37 °C. Unstimulated PBMCs were stained for surface markers using validated, published methods^22–24^. Cells were acquired on a Helios mass cytometer (Fluidigm) followed by concatenation and normalization of raw data according to the manufacturer’s instructions. Computational approaches were utilized on bead normalized data files gated for live singlets using the t-SNE algorithm to analyze and visualize the multidimensional data in two dimensions as unsupervised clustering (viSNE, Cytobank). Supplemental Fig. S2 illustrates the CyTOF workflow from PBMCs through data analysis.

**Figure 1:**
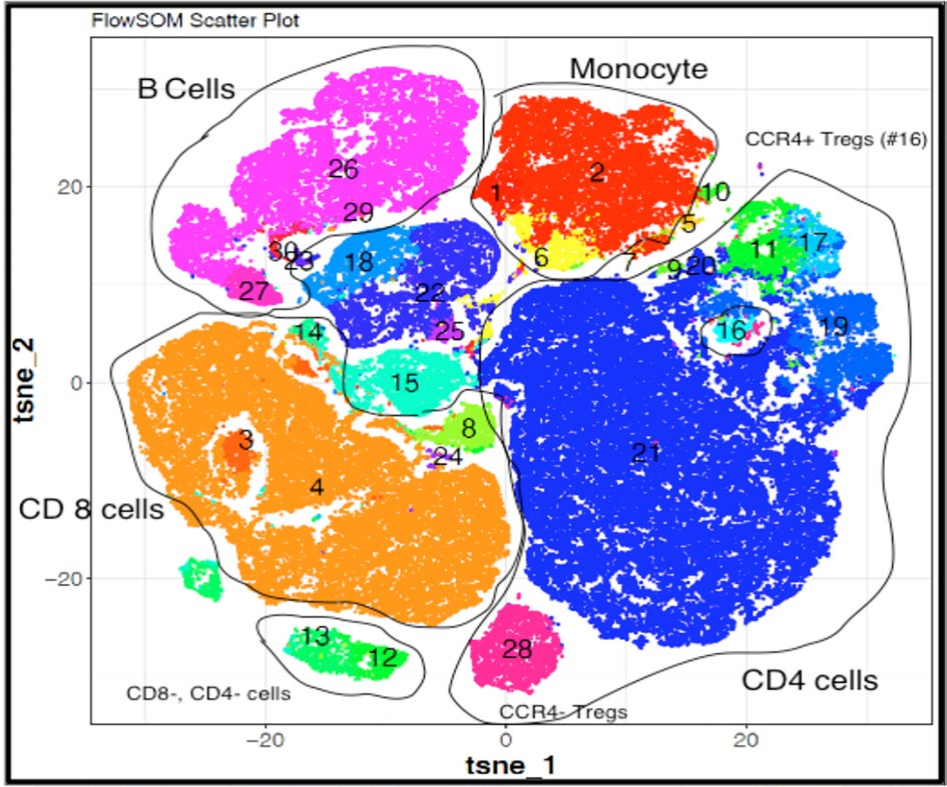
CyTOFkit software generated viSNE scatterplots show a distinguished immune cell architecture of PBMCs isolated from minority children living in polluted areas. Cell types are shown in different colors representing intensity of their corresponding surface markers (tsne: t-distributed stochastic neighbor embedding, n = 191). Details on the cluster identification by cell type and surface markers can be found in Supplemental Table S4.

CyTOF FlowSOM clusters of immune cells were visualized using a non-linear dimensionality reduction algorithm termed visualization of t-distributed stochastic neighbor embedding (viSNE), shown in Fig. 1 with the corresponding surface cell markers indicating dominant clusters of CD8+ (orange) and CD4+ (blue), B cells (purple) and monocytes (red). Supplemental Fig. S3 shows subsets further refined in the form of a heat map based on relative expression levels of CyTOF markers from the FlowSOM metaclusters from least (blue) to most (red) intensity. Cell clusters were manually annotated based on the expression levels of markers and indicates the highest intensity (expression) of cell surface markers were concentrated as CD4 + T cells (cluster 21: CD3+, CD4+), CD8 + T cells (cluster 4: CD3+, CD8a+), monocytes (cluster 2: CD14+,CCR5+, CD 161+) and B cells (cluster 26: CD20+,CD196+). The major immune cell clusters and surface markers are listed in Supplemental Tables S3 and S4 presents the mean MFI and standard deviations for each cluster for all subjects in the cohort.

### Statistical analyses

CyTOF was performed for cellular identification. Based on prior data our sample size calculation estimated a minimum of 100 samples (50 in each group) would be sufficient to detect a 20% change in immune cells using this method. After the CyTOF data was normalized, a t-Distributed Stochastic Neighbor Embedding (t-SNE) algorithm was run to determine immune cell clusters in an unsupervised manner using FlowSOM by R 3.6.0 programming. viSNE plots were also analyzed in the same manner^25^.

Using JMP Genomics v6.0, we applied partial least squares (PLS) to identify patterns of average air pollution exposures linked with methylation of immunoregulatory genes, immune cell typology and BP levels. We chose this multivariate method due to its ability to deal with large sets of highly correlated predictors (e.g. air pollutants) and outcomes (e.g. methylation sites). PLS creates linear combinations (latent factors) of the predictors (average air pollutant exposures) so that the covariance between the predictors and the outcome variables (methylation, immune cell typology or elevated BP) is maximized. These latent factors are then used instead of the original individual predictors for outcome prediction. Per outcome, we selected the PLS model with the most optimal number of latent factors (which predicted the outcome best at balanced risk for under- and overfitting), as the model with the lowest predicted residuals sum of squares (PRESS) value explaining a substantial proportion of variation in both predictor and outcome variables. While PLS is a linear regression model, it differs from the classical multiple linear regression approach in that only the relevant predictors identified by the latent factors were used to predict outcome. The importance of each predictor in the construction of the latent factors was determined from the variable importance in projection (VIP) scores of Wold. Higher VIP score implies a higher relevance of the predictor to predict the response variable. In our analysis, predictors with a VIP > 1.3 were considered influential and were investigated in more detail.

To validate the PLS findings, we compared the multivariable-adjusted associations between the air pollutants identified in PLS and the outcome of interest (immunoregulatory gene methylation, immune cell typology and/or BP levels) using R software version 3.3.1^26^, while accounting for age, sex, race and BMI. The linear regression models additionally accounted for asthma status, given the high percentage of asthmatic children (23%) in this cohort and its possible comorbidity with hypertension. Supplemental Table S5 compares the cell types, methylation percentage and blood pressure between non-asthmatic and asthmatic subjects and there were no significant differences. It should be noted that the directionality of the relationships between one of the air pollutants and one of the outcomes can differ between PLS and the linear regression models. Such differences in directionality originate from the methodological differences between both approaches. Whereas PLS is capable of dealing with vast and complex networks of interrelated predictors and outcomes, multiple linear regression could only be applied to relatively small sets of predictors as to avoid disruption of the models due to collinearity.

## Results

### Characteristics of study population

Table 1 presents the characteristics of the study population. There were 221 children (46.2% female, 53.8% male) with a median age of 7.9 years (7.6–8.4). Most of the enrolled children were Hispanic (77%). Mean systolic BP was 106.1 ± 10.97 mmHg and mean diastolic BP was 64.9 ± 8.18 mmHg.

**Table 1:** Demographic characteristics of child cohort.

Characteristic	Total Cohort (n = 221)
Age, years	7.9 ± .3
Female, n (%)	102 (46%)
BMI percentile	72 ± 26.9
Systolic BP, mmHg	106.1 ± 11.0
Diastolic BP, mmHg	64.9 ± 8.2
Asthma, n (%)	51 (23%)
Rhinitis, n (%)	15 (7%)
Eczema, n (%)	34 (15%)
Food allergy to any of 8 major allergens, n (%)	10 (5%)
Secondhand smoke exposure in home, n (%)	36 (16%)
**Household income**	
< $15,000, n (%)	68 (31%)
$15,000–$30,000, n (%)	82 (37%)
$31,000–$50,000, n (%)	43 (20%)
$51,000–$75,000, n (%)	19 (8%)
$76,000–$100,000, n (%)	5 (2%)
> $100,000, n (%)	2 (1%)
**Race/ethnicity**	
Hispanic, n (%)	170 (77%)
African American, n (%)	30 (14%)
White, n (%)	13 (6%)
Asian/Pacific Islander, n (%)	8 (4%)
Participated in warm season (April–Sept), n (%)	116 (53%)
Participated in Cold season (Oct–March), n (%)	105 (48%)

Values are mean ± SD and number (%).

*BMI percentile was calculated from the Z scores per the CDC growth chart recommendations.

### Air pollution is linked with methylation of immunoregulatory genes

**Figure 2:**
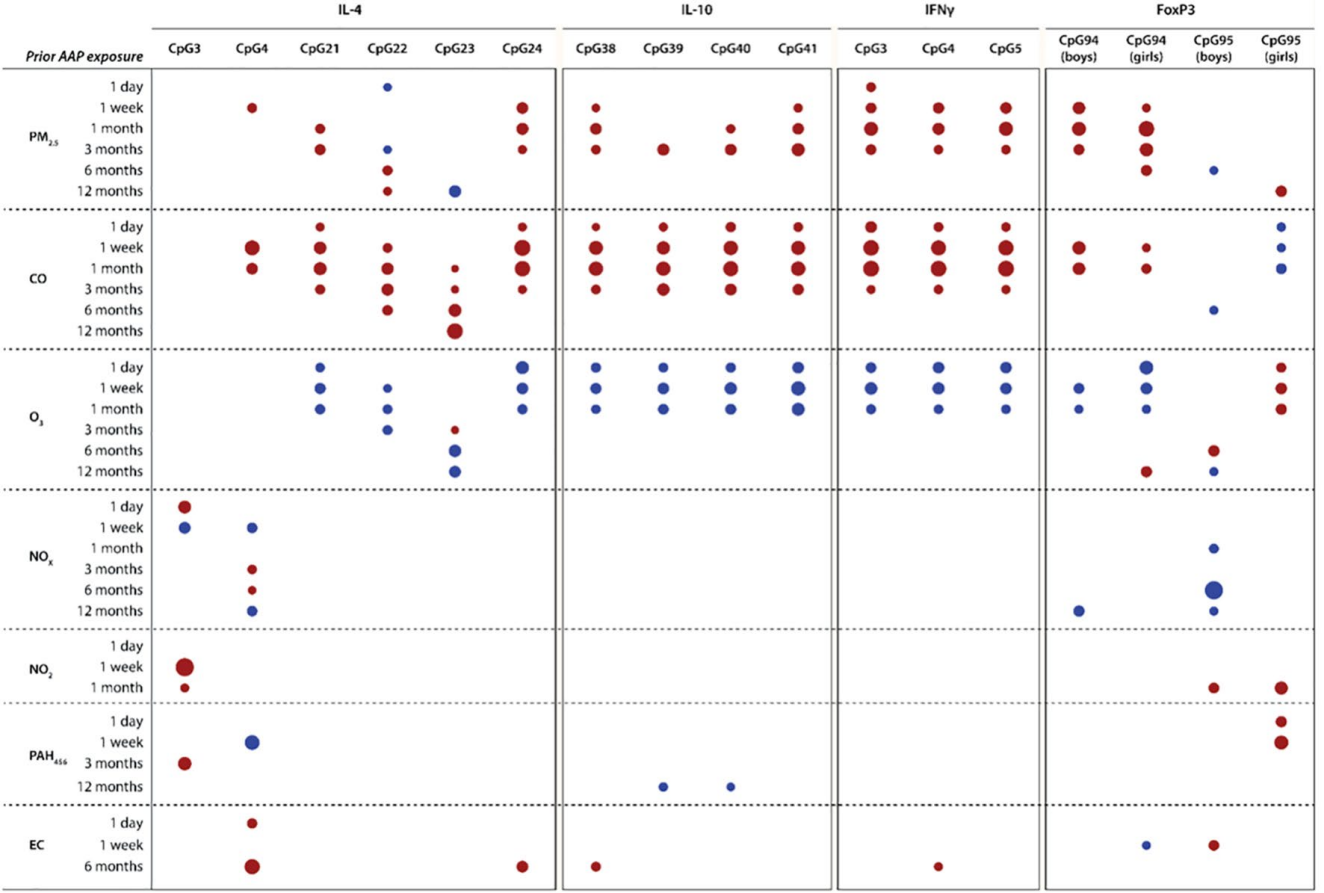
CpG Site Methylation of Immunoregulatory Genes and Prior Ambient Air Pollution (AAP) Exposures. Heat map shows the air pollutant averages by exposure time predicting methylation of the IL-4, IL-10, IFNg and FoxP3 gene in PLS modeling. Red are positive and blue are negative correlations. The size of the marker reflects the VIP value (VIP > 1.3 for all). Supplemental Table S6 presents the summary data for each PLS model. *PLS* partial least squares.

We first performed multivariate PLS to identify the main predictors (pollution averages) and outcomes (methylation sites) that were most likely to be related and found that both short and long-term exposure to PM_2.5_ (1 to 3 months prior), CO (1 day to 12 months prior) and O_3_ (1 day to 6 months prior) were key variables responsible for predicting most of the immunoregulatory gene methylation (see Table 2). Because the Foxp3 gene is on the X chromosome, we analyzed the Foxp3 CpG sites separately for males and female. Figure 2 summarizes the influential air pollutant averages by exposure time predicting methylation for each immunoregulatory CpG site studied. Consistent with using methylation site averages, the PLS models per CpG site suggested associations between recent (< 6 months) exposure to PM_2.5_, CO and ozone and most of the methylation sites except IL-4 CpG3 and CpG4 sites and the FoxP3 CpG95 site (in boys). Supplemental Table S6 presents the summary data for each PLS model, in which the models explained between 6.9 and 50.4% of the variation in CpG sites’ methylation.

**Table 2:** Multivariate partial least squares modeling to identify key air pollutant exposures predicting key CpG methylation sites of immunoregulatory genes.

	CpG methylation of IL-4, IL-10, IFNγ and FoxP3
Number of latent factors	2
**% of variation explained by latent factors**	
In predictors (air pollutants)	37.8
In outcomes (methylation)	12.3
Key variables responsible for outcome prediction (VIP > 1.3)	**CO exposure** (1 day to 12 months prior) **O**_**3**_** exposure** (1 day to 6 months prior) **PM**_**2.5**_** exposure** (1 to 3 months prior)
Key predicted outcome markers	All CpG sites, excl. FoxP3—CpG island 95 and IL-4—CpG islands 3 and 4

Air pollutant exposures included 1 day to 12 month averages for PM_2.5_, carbon monoxide (CO), ozone (O_3_), nitrogen oxides (NO_X_ and NO_2_), polycyclic aromatic hydrocarbons (PAH_456_) and elemental carbon. VIP indicates variable importance in projection.

Supplemental Table S7 shows the multivariable-adjusted associations between the CpG methylation sites and the air pollution exposures preselected in PLS. When performing multivariable-adjusted regression analyses, CpG methylation of the immunoregulatory genes was independently associated with most of the air pollution exposures highlighted in the PLS analyses. For example, higher PM_2.5_ exposure 1 month prior to the study visit was independently associated with greater methylation of the IL-4 CpG sites 21 and 24, IL-10 CpG sites 38, 40 and 41, IFNγ CpG sites 3 to 5 and the FoxP3 CpG94 site (standardized estimates (est) between 0.13 and 0.38, *P* ≤ 0.040 for all). Furthermore, methylation of the IL-4 CpG sites 21 to 24, the IL-10 CpG sites 38 to 41 and the IFNγ CpG sites 3 to 5 was associated with higher one-month prior CO exposure (est between 0.25 and 0.41, *P* < 0.0001 for all). In addition, after full adjustment, acute (≤ 1 month) O_3_ exposure was inversely associated with gene methylation of IL-4 (CpG sites 21, 22 and 24), IL-10 (CpG sites 38 to 41) and IFNγ (CpG sites 3 to 5) (est between − 0.17 and − 0.43, P ≤ 0.0017) and directly associated with Foxp3 CpG95 site methylation in girls (est between 0.31 and 0.34; P ≤ 0.0001).

### Air pollution is linked with dysregulated immune cell profiles

In multivariate PLS, recent exposure to PM_2.5,_ CO, O_3_, and PAH_456_ were main predictors of the immune cell profiles, particularly of Th1, Th2 and Th17 cell percentages (see Table 3; Supplemental Fig. S4). PLS explained only 3.5% of the variance in the immune cell types, likely due to the heterogeneous outcomes. Indeed, when relaunching PLS with only Th1, Th2, Th17 and T regulatory cells as outcomes, the same key predictors were identified (recent exposure to PM_2.5_, CO, O_3_, CO and PAH_456_) but with much higher variance of outcome explained (9%).

**Table 3:** Multivariate partial least squares modeling to identify key air pollutant exposures predicting key immune cell typology.

	Immune cell typology
Number of latent factors	1
**% of variation explained by latent factors**	
For predictors (air pollutants)	26.7
For outcome (immune cells)	3.5
Key variables responsible for outcome prediction (VIP > 1.3)	**CO exposure** (1 day to 3 months prior) **O**_**3**_** exposure** (1 day to 1 month prior) **PM**_**2.5**_** exposure** (1 week to 3 months prior) **PAH**_**456**_** exposure** (1 day to 1 month prior)
Key predicted outcome markers	Th1, Th2 and Th17 cells

Air pollutant exposures included 1 day to 12 month averages for PM_2.5_, carbon monoxide (CO), ozone (O_3_), nitrogen oxides (NO_X_ and NO_2_), polycyclic aromatic hydrocarbons (PAH_456_) and elemental carbon. Th indicates T helper; VIP, variable importance in projection.

**Figure 3:**
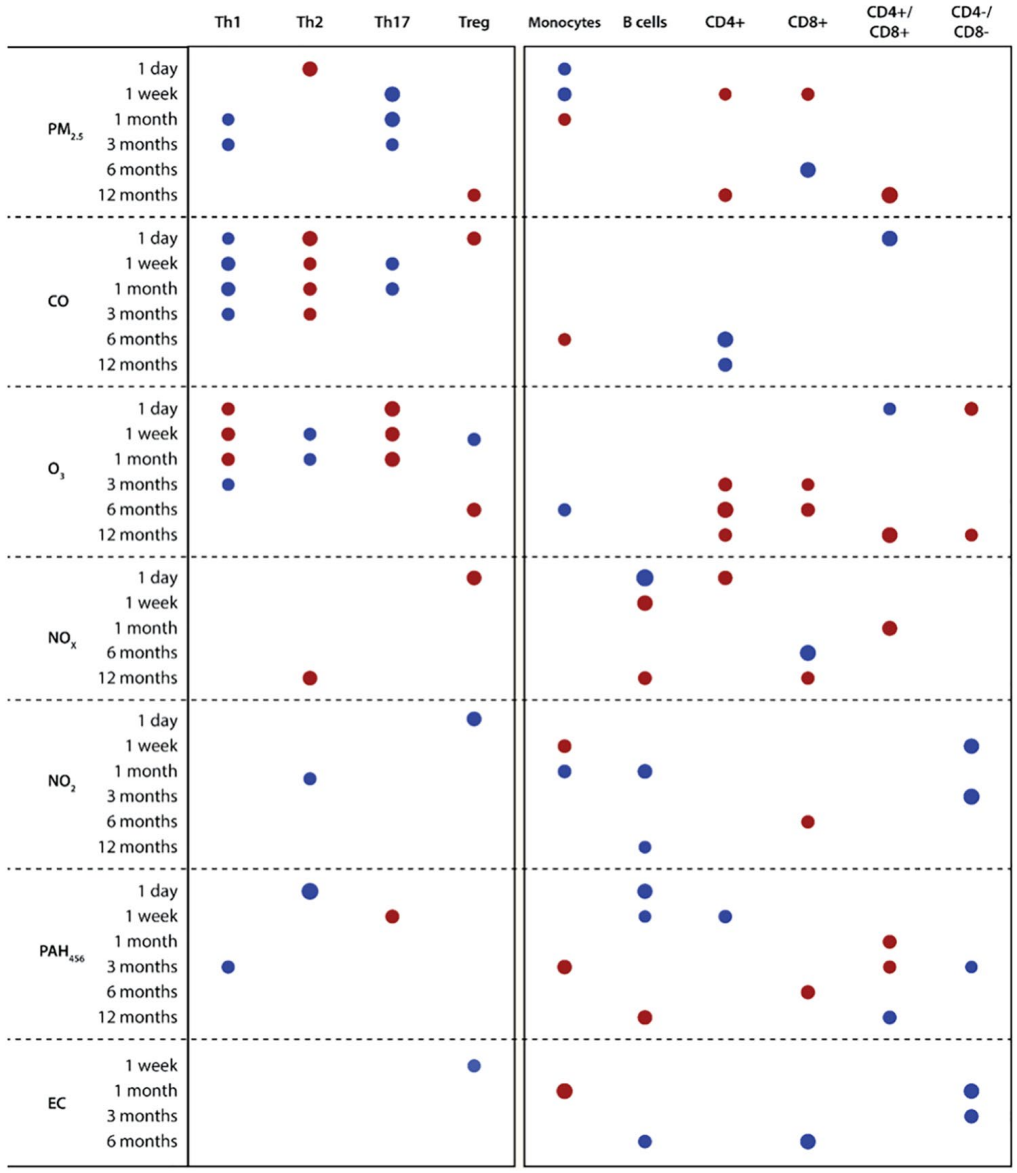
Immune cell typology and prior ambient air pollution exposures. Heat map shows the air pollutant averages by exposure time predicting immune cell typology in PLS modeling. Red are positive and blue are negative correlations. The size of the marker reflects the VIP value (VIP > 1.3 for all). Summary data for each PLS model can be found in Supplemental Table S8. *PLS* partial least squares.

Next, we constructed PLS models for each immune cell type separately. Figure 3 summarizes the influential air pollutant averages by exposure time per immune cell type. Consistent with the multivariate model, the PLS models per immune cell type suggested associations between recent (< 6 months) exposure to PM_2.5_, CO and ozone and Th1, Th2 and Th17 cell percentages. Supplemental Table S8 presents the results, with air pollution explaining between 4.3 and 33.6% of the variation in the immune cell profiles.

Supplemental Table S9 shows the multivariable-adjusted associations between the immune cell types and the air pollution exposures preselected in PLS analyses. In multivariable-adjusted analyses, we found significant associations of Th1 and Th17 cells with 1 week to 3 month exposure to PM_2.5_ (est − 0.14 to − 0.34), CO (est − 0.16 to − 0.44) and O_3_ (est 0.19 to 0.44; P ≤ 0.026 for all). Furthermore, higher Th2 cell percentage was associated with higher 1 day PM_2.5_ exposure, higher 1 day to 3 month CO exposure, lower 1 week to 1 month O_3_ exposure, lower 3 month NO_2_ exposure and lower 1 day PAH_456_ exposure (P ≤ 0.033 for all), In contrast, the air pollution exposures selected in PLS were not associated with Treg cell percentage in multiple linear regression analyses (Supplemental Table S9). In addition, we observed significant associations between the monocyte cell percentage and 1 month exposures to PM_2.5_ (est = 0.17, P = 0.025) and CO (est = − 0.20, P = 0.0081) (Supplemental Table S9).

We used linear regression to confirm the PLS-predictions of the association between B cells, CD8 + cells and CD4 + cells (not further identified) with NO_2_, NOx and PAH_456_ (Supplemental Table S9)_._ Both B cells (est = − 0.19) and CD4 + cells (est = 0.16) were associated with 1 day NO2 exposure (P ≤ 0.031). Furthermore, CD4 + and CD8 + but not B cells were associated with chronic long-term prior pollution exposure: CD8 + cells at 6 months exposure to PAH_456_ (est = 0.16) and NOx (est = − 0.20) and CD4 + cells at 6 months exposure for CO (est = − 0.22) and NO_2_ (est = − 0.26)(*P* ≤ 0.038 for all).

### Air pollution is linked with changes in blood pressure

For prediction of BP levels, PLS models incorporating the air pollution exposures explained 21.0% and 5.1% of the variance in systolic and diastolic BP, respectively, and 7.0% of the variance in pulse pressure. Table 4 presents the summary data for the PLS models and highlights the top pollutants responsible for BP level prediction. When performing the multivariable-adjusted linear regression, higher systolic BP was independently associated with higher 3-month exposure to NOx, 1-day exposure to NO_2_ and 6-month exposure to PAH_456_ (est between 0.13 and 0.17; P ≤ 0.043; Supplemental Table S10).

**Table 4:** Blood Pressure (BP) levels and prior ambient air pollution exposure.

	Systolic BP	Diastolic BP	Pulse pressure
Number of latent factors	3	1	1
**% of variation explained by latent factors**			
For predictors (air pollutants)	38.2	20.4	22.7
For outcome (BP level)	21.0	5.1	7.0
Top predictors responsible for outcome (VIP > 1.3)	Positive correlation: NO_X_ (3 month) NO_2_ (1 day) PAH_456_ (1 week, 6 month) Negative correlation: O_3_ (12 month) PAH_456_ (1 month)	Positive correlation: NO_X_ (1 week) PAH_456_ (6 month) Negative correlation: PM_2.5_ (3 and 6 month) CO (3 m and 6 month) NO_X_ (1 month) PAH_456_ (1 month) EC (1 month)	Positive correlation: NO_2_ (1 day) NO_X_ (3 month) Negative correlation: PM_2.5_ (3 month) O_3_ (1 and 12 month) CO (3 and 12 month) NO_X_ (12 month)

The table present the summary data for the partial least squares models predicting systolic and diastolic BP and pulse pressure from air pollutant exposures (including 1 day to 12 month averages for PM_2.5_, carbon monoxide (CO), ozone (O_3_), nitrogen oxides (NO_X_ and NO_2_), polycyclic aromatic hydrocarbons (PAH_456_) and elemental carbon (EC). VIP, variable importance in projection.

Diastolic BP was associated with long-term (6 month) exposure to PM_2.5_, CO and PAH_456_ (est − 0.13, − 0.17 and 0.17, respectively, *P* ≤ 0.020), but not with short-term exposure to NOx or PAH_456_ (Supplemental Table S10).

### Monocytes are linked with systolic blood pressure

We performed PLS for prediction of BP levels by immune cell types and CpG methylation sites of immunoregulatory genes. In both unadjusted and adjusted PLS analyses, key immune cell types and methylation sites for systolic BP were CD8 + cells, monocytes and methylation of IL-10 (CpG island 41) and IL-4 (CpG island 22). In multivariable-adjusted linear regression, only monocytes were significantly associated with systolic BP (est = 2.39; *P* = 0.02). In PLS, Th17 and methylation of FoxP3 (CpG islands 94 and 95) and IL-10 (CpG island 38) were identified as influential markers for prediction of diastolic BP, but none of these were related to diastolic BP in multivariable-adjusted linear regression (*P* > 0.30 for all).

## Discussion

We found associations between air pollution exposure and methylation of immunoregulatory genes, protein expression of associated immune cell types and clinical expression of blood pressure. This study thus suggests that pollution exposure early in life may be associated with multiple outcomes, potentially impacting long-term health into adulthood.

Our findings show that ambient air pollution exposure is associated with methylation of immune-regulatory genes, which is consistent with and expands previous research^27^. The effects of air pollution on the immune and cardiovascular system are thought to be mediated by oxidative stress, apoptosis, inflammation, and immune-mediated injury, while releasing proinflammatory and vasoactive factors contribute to cardiopulmonary pathology^5,28–32^. Here we demonstrated air pollution as a predictor of DNA methylation of 4 immunoregulatory genes: IL-4, IL-10, Foxp3 and IFNγ. Overall, acute exposure to CO, O_3_ and PM_2.5_ was associated with methylation of numerous CpG sites. The largest effect size (B estimate = 4.7) was for the association of CO levels at 15 days prior to blood draw to a CpG site in the promoter region of the IL-10 gene. We interpreted a change in CpG site methylation as a percentage, such that if Child A’s average exposure to CO over the 15 days before the clinic visit was 1 µg/m^3^ higher than Child B, Child A would be expected to have increased methylation of the IL10 gene at that CpG site which is 4.7% higher than Child B. We previously found that air pollution exposure in adolescents was associated with epigenetic changes in the Foxp3 gene, a transcription factor for T regulatory cells, and worsening asthma^13,33^ Cardiometabolic outcomes have also been associated with air pollution exposure and DNA methylation^34,35^. Epigenetic modifications such as methylation results in altered regulation of protein expression. Therefore, we used CyTOF to measure immune cell expression to associate the cellular expression with pollution exposure on unsorted cells, as we have found that cell type composition does not substantially affect DNA methylation variability^32^.

Overall, our findings showed that monocytes and T helper cell types (Th1, Th2 and T regulatory) are impacted by acute exposure to air pollution (e.g. O_3_, CO and PM_2.5_). In addition, B cells, CD8 + cells and not further identified CD4 + cells were associated with both acute and chronic exposure to NO_2_, NOx and PAH_456_. The effect sizes were relatively small, with the largest effect size found for monocytes levels and acute exposure to CO at 1 month (absolute estimate = − 1.12). Thus, a 1 ppm increase in average CO exposure over 1 month prior to blood draw was associated with a 1% decrease in monocyte cell expression. This may represent a transformation of monocytes in the blood to tissue macrophages in the lung and inflammatory dendritic cells in skin, especially given that PM_2.5_ increases permeability and transmigration of monocytes across endothelial monolayer^36^. Studies have found that intratracheal administration of mice with soot, PM_1_, or PM_10_ increased recruitment of both macrophages and dendritic cells into the airways^37^ and Gawda et al. also recently reported that stimulation with PM induced secretion of pro-inflammatory cytokines and primed monocytes and macrophages to a hyperinflammatory response in vitro^38^. Consistent with our findings, a group of preschoolers exposed to PAH and lead near an e-waste facility in China had elevated monocytes and CD 4 + cells, but not CD 8 + cells^39^ and Becker et al. reported that exposure to urban air particulates decreased monocytes^40^. However, other studies found inconsistent results with monocyte enrichment and DNA methylation in monocytes after long-term exposure to air pollution^41^.

Blood pressure was also associated with air pollution, although overall the results were not as consistent as the methylation findings. Diastolic BP was inversely associated with long-term exposure to CO and directly with long-term exposure to PAH_456_. In addition, acute exposure to CO (3 months) had a significant absolute effect size of -7.25, which can be interpreted as if Child A’s average acute exposure to CO prior to the clinic visit was 1 µg/m^3^ higher than Child B, Child A would be expected to have a diastolic BP 7.25 mmHg lower than Child B. This inverse relationship may be related to the increased ventricular compliance and distensibility in younger individuals^42^. For pulse pressure, PM_2.5_ at 3 months had an effect size of 0.58, which can be interpreted as if Child A’s average exposure to PM_2.5_ at 3 months prior to the clinic visit was 1 µg/m^3^ higher than Child B, Child A would be expected to have a pulse pressure 0.58 higher than Child B. In adults, the most important cause of elevated pulse pressure (the difference between the systolic and diastolic BPs) is stiffness of the aorta and these results support a study that found that even in 5 year olds, air pollution exposure may reduce arterial distensibility^43^. Our systolic blood pressure findings were the least convincing overall, as some studies have found associations. For example, Pieters et al. reported children (6–12 years) with same-day exposure to nano-sized PM (20–30 nm) had an association with increased systolic but not diastolic BP^8^ and children exposed to ultrafine PM or PM_2.5_ in combination with NO_2_ had elevated systolic and diastolic blood pressures^8,9^. In general, the air pollution-blood pressure literature has inconsistencies, especially in young children. This has been attributed to variations in study design, population characteristics, exposure duration, air pollutant concentrations, exposure measures, BP measurements^44^, and it has even been proposed that breastfeeding (which we did not control for) may reduce this association^45^. Follow-up studies are needed to determine whether these BP findings can be replicated.

Interestingly, systolic BP was also linked with monocyte expression. Specifically, we found that 1 mmHg increase in systolic pressure was associated with a 2.4% increase in monocyte levels. This is compelling because monocytes play a central role in inflammasome activation and cardiovascular disease^46,47^ and the differentiated macrophage is the most abundant cell in atherosclerotic plaques^48,49^. Importantly, monocytes from hypertensive versus healthy individuals show significant increases in IL-1β and TNF-α secretion, indicating monocytes are preactivated in patients with increased blood pressure^50^ and compared with normal individuals, patients with essential hypertension show a significant increase in monocyte migration^51^, which is an early step in the atherosclerotic process^52^.

Whereas most air pollution health studies have focused on adults, some of which already have overt clinical cardiac disease, a strength of our study was the inclusion of participants in a young age group, allowing us to investigate air pollution effects on cardiovascular and immune functioning before clinical symptomatology. The Fresno cohorts have been previously studied and are well-characterized using rigorous and validated measures previously described^13,15,33^. The children in our study were primarily Hispanic (77%), representing the largest and rapidly growing minority group of youth in the United States and experience more health disparities than non-Hispanic white children^53^, especially in California where they are exposed to higher traffic-related pollution levels than non-Hispanic children^54^. Moreover, prevalence for uncontrolled hypertension is greater for Hispanic adults compared with other races and ethnicities in the U.S.^55^, increasing the importance to determine preclinical risk as children exposed to elevated air pollution progresses into adulthood. Another strength was our cross-sectional design, measuring the prevalence for several pollution endpoints over time, and allowing an analysis of how various air pollution exposure levels and pollution types (most studies to date have focused on a single pollutant type). Cross-sectional studies have been used to determine major findings in the field of air pollution and health^56,57^, with a large population-based study demonstrated significant associations between air pollution and stroke and CVDs in adults^58^. Finally, this is the first time that CyTOF was utilized to immunophenotype cells in a pollution study. CyTOF is superior in both data acquisition and biostatistical analysis than traditional flow cytometry^59^, and enable us to simultaneously analyze many immune cell markers simultaneously.

Our pollution estimates were based on the levels outside the children’s’ homes. However, most studies of air pollution health effects have used similar methods because personal exposure monitoring to measure both indoor and outdoor exposures is not feasible for long study periods. In addition, exposure averages at 12 months were less reliable because of the small exposure contrast over this time period in a single city. Also, clinical outcomes were generally based on parental reports of a physician’s previous diagnosis of their children, and a cross-sectional analysis did not allow us to examine multiple BP measurements over time because hypertension is clinically diagnosed as elevated BP readings (> 130/80) across 3 timepoints. A study of longer duration would have provided a more comprehensive picture of the effects of air pollution on children’s health. Lastly, our two-step statistical approach of PLS feature selection followed by ordinary least squares for inference may have inflated reported *P* values through overfitting, making the multilinear regression models exploratory.

In conclusion, we find that air pollution exposure is linked to methylation of immunoregulatory genes, immune cell profiles and blood pressure, suggesting that even at a young age, the immune and cardiovascular systems are negatively impacted by air pollution.

## Supplementary Information

Supplementary Information.
