# Supplementary material for: Air pollution exposure is linked with methylation of immunoregulatory genes, altered immune cell profiles, and increased blood pressure in children
Source: Sci Rep. 2021 Feb 18;11:4067. doi: 10.1038/s41598-021-83577-3 (PMC7893154; doi:10.1038/s41598-021-83577-3)
Supplement: Supplementary file 1 — Supplementary Information. [file 41598_2021_83577_MOESM1_ESM.docx]

**SUPPLEMENTARY TABLES AND FIGURES**

**Air pollution exposure is linked with methylation of immunoregulatory genes, altered immune cell profiles, and increased blood pressure in children**

Mary Prunicki, PhD, MD^1,3^, Nicholas Cauwenberghs, PhD^2^, Justin Lee, MPH^1,4^, Xiaoying Zhou, PhD^1,3^, Hesam Movassagh, PhD^1.3^, Elizabeth Noth, PhD^5^, Fred Lurmann, MS^6^, S. Katharine Hammond, PhD^5^, John R. Balmes, MD^5,7^, Manisha Desai, PhD^4^, Joseph C. Wu, MD, PhD^3,8^ and Kari C. Nadeau, MD, PhD^1,3^*

**Affiliations:**

^1^Sean N. Parker Center for Allergy and Asthma Research at Stanford University, CA 94305 USA

^2^Department of Cardiovascular Sciences, University of Leuven, Belgium

^3^Department of Medicine, Stanford University, Stanford, CA 94305 USA

^4^Quantitative Sciences Unit, Stanford University, Stanford, CA 94305 USA

^5^School of Public Health, University of California, Berkeley, Berkeley, CA 94720, USA

^6^Sonoma Technology, Inc., Petaluma, California 94954, USA

^7^Department of Medicine, University of California, San Francisco, CA 94143, USA

^8^Stanford Cardiovascular Institute, Stanford University, Stanford, CA 94305 USA

*Corresponding Author: Kari C. Nadeau, MD, PhD, Sean N. Parker Center for Allergy and Asthma Research at Stanford University, Division of Pulmonary and Critical Care Medicine, Department of Medicine, Stanford University, Stanford University School of Medicine, 269 Campus Drive, CCSR 3215, MC 5366, Stanford, CA 94305-5101, USA. E-mail: [knadeau@stanford.edu](mailto:knadeau@stanford.edu).

**Supplementary Materials**

**Table S1:** The mean percent methylation and standard deviation for each CpG site of the 4 genes Foxp3, Il-4, Il-10 and IFN-γ for all subjects in the cohort.

| **Gene** | **CpG site** | **Mean (Standard Deviation)** |
| --- | --- | --- |
| Foxp3 | 94 | 46.36 (4.64) |
|  | 95 | 63.28 (6.15) |
| Il-4 | 3 | 83.24 (3.45) |
|  | 4 | 85.84 (12.73) |
|  | 21 | 82.83 (3.35) |
|  | 22 | 75.66 (6.17) |
|  | Loss 23 | 59.89 (12.0) |
|  | 24 | 51.19 (5.39) |
| Il-10 | 38 | 64.70 (6.63) |
|  | 39 | 46.55 (7.62) |
|  | 40 | 53.77 (7.67) |
|  | 41 | 33.95 (9.18) |
| IFNγ | 3 | 70.55 (6.86) |
|  | 4 | 64.31 (6.99) |
|  | 5 | 68.62 (7.16) |

**Table S2:** CyTOF panel. Antibodies were obtained from Fluidigm

(South San Francisco, CA, USA).

| **Catalog #** | **Metal** | **Chemical element** | **Marker** |
| --- | --- | --- | --- |
| 3141003A | 141 | Pr | CD196 (CCR6) |
| 3145001B | 145 | Nd | CD4 |
| 3146001B | 146 | Nd | CD8a |
| 3147001B | 147 | Sm | CD20 |
| 3148010B | 148 | Nd | CD14 |
| 3149010B | 149 | Sm | CD25 |
| 3153013A | 153 | Eu | CCR4 |
| 3154003B | 154 | Sm | CD3 |
| 3156015A | 156 | Gd | CCR5 (CD195) |
| 3163004B | 163 | Dy | CXCR3 (CD183) |
| 3164009B | 164 | Dy | CD161 |
| 3176004B | 176 | Yb | CD127 (IL-7R) |

**Table s3.** Cluster Identification by Cell Type and Surface Markers

| **Cell Type** | **Cluster Numbers** | **Cluster Surface Marker** |
| --- | --- | --- |
| Monocytes (CD14+) | 1,2,6 | 1: CD161+  2: CCR5+, CD161+  6: CCR5+ |
| B cells (CD20+) | 26, 27 | 26: CD196+  27: CXCR3+, CCR4+, CD196+ |
| CD8+ cells  (CD3+, CD8a+) | 3, 8, 14 | 3 : CXCR3+, CCR5+  8: CD161+, CD127+, CD196+  14: CXCR3+, CCR4+, CD196+ |
| CD8+ cells  (CD3+, CD8a+) | 4 | Quantity suggests naïve population.  (Memory marker not used) |
| Th 1 cells  (CD3+,CD4+) | 9 | CXCR3+, CCR5+ |
| Th 2 cells  (CD3+, CD4+) | 19 | CCR4+, CCR6- |
| T regulatory cells  (CD3+, CD4+) | 16, 28 | 16: CD25+, CD127-  28: CD25+, CD127- |
| Th 17 cells  (CD3+, CD4+) | 17 | CCR4+, CCR6+ |
| CD 4+ cells  (CD3+, CD4+) | 21 | 21: Quantity suggests naïve population.  (Memory marker not used) |
| CD 4+ cells  (CD3+, CD4+) | 11 | CD161+, CD127+  (Probable effector or memory cells) |
| CD4+ cells  (CD3+, CD4+) | 20 | CD196+, CXCR3+, CCR4+ |
| Double positive T cells  (CD4+/CD8a+) | 24 | CD127+ |
| CD4-/CD8- cells  (CD3+) | 12, 13, 15 | 12: CCR5+,CD127+, CD161+, CD196+  13:CXCR3+, CCR5+,CD127+, CD161+, CD196+  15: CXCR3+, CD127+ |
| Unidentified Clusters | 5,7,10,18,22,  23,25,29,30 | Not applicable |

**Table s4.** Mean MFI and standard deviation for each cluster for all subjects in the cohort.

| **Cell Type** | **Cluster Numbers** | **Mean (Standard Deviation)** |
| --- | --- | --- |
| Monocytes (CD14+) | 1,2,6 | Cluster 1: 0.65 (0.69)  Cluster 2: 8.42 (3.99)  Cluster 6: 1.06 (0.96) |
| B cells (CD20+) | 26, 27 | Cluster 26: 9.86 (3.78)  Cluster 27: 0.75 (0.53) |
| CD8+ cells  (CD3+, CD8a+) | 3, 8, 14 | Cluster 3: 0.83 (0.78)  Cluster 8: 1.22 (1.11)  Cluster 14: 0.30 (0.33) |
| CD8+ cells  (CD3+, CD8a+) | 4 | Cluster 4: 21.5 (5.61) |
| Th 1 cells  (CD3+,CD4+) | 9 | Cluster 9: 0.25 (0.27) |
| Th 2 cells  (CD3+, CD4+) | 19 | Cluster 19: 3.33 (1.48) |
| T regulatory cells  (CD3+, CD4+) | 16, 28 | Cluster 16: 0.46 (0.34)  Cluster 28: 2.38 (1.03) |
| Th 17 cells  (CD3+, CD4+) | 17 | Cluster 17: 0.85 (0.65) |
| CD 4+ cells  (CD3+, CD4+) | 21 | Cluster 21: 32.66 (8.29) |
| CD 4+ cells  (CD3+, CD4+) | 11 | Cluster 4: 1.57 (0.88) |
| CD4+ cells  (CD3+, CD4+) | 20 | Cluster 20: 0.30 (0.34) |
| Double positive T cells  (CD4+/CD8a+) | 24 | Cluster 24: 0.16 (0.21) |
| CD4-/CD8- cells  (CD3+) | 12, 13, 15 | Cluster 12: 0.75 (0.97)  Cluster 13: 1.33 (1.24)  Cluster 15: 3.11 (1.44) |

**Table s5.** Comparison of non-asthmatic and asthmatic subjects for cell type, methylation percentage and blood pressure.

| **Cell Type** | **Non-Asthmatic Mean MFI** | **Asthmatic**  **Mean MFI** | **P value** |
| --- | --- | --- | --- |
| Th1 | 3.36 | 3.59 | 0.49 |
| Th2 | 7.59 | 7.87 | 0.61 |
| Th17 | 3.68 | 4.25 | 0.11 |
| T regulatory | 6.73 | 6.62 | 0.70 |
| **Gene/CpG site** | **% methylation** | **% methylation** |  |
| Foxp3 / 94 | 46.28 | 46.67 | 0.58 |
| Foxp3 / 95 | 63.31 | 63.20 | 0.90 |
| Il-4 / 3 | 83.05 | 84.00 | 0.09 |
| Il-4 / 4 | 85.30 | 87.90 | 0.20 |
| Il-4 / 21 | 82.88 | 82.62 | 0.61 |
| Il-4 / 22 | 75.78 | 75.11 | 0.68 |
| Il-4 / loss 23 | 60.47 | 57.72 | 0.13 |
| Il-4 / 24 | 51.28 | 50.87 | 0.61 |
| Il-10 / 38 | 64.89 | 63.97 | 0.35 |
| Il-10 / 39 | 46.50 | 46.76 | 0.83 |
| Il-10 / 40 | 53.77 | 53.75 | 0.99 |
| Il-10 / 41 | 33.93 | 34.04 | 0.94 |
| IFNγ / 3 | 70.72 | 69.92 | 0.44 |
| IFNγ / 4 | 64.48 | 63.68 | 0.44 |
| IFNγ / 5 | 68.72 | 68.24 | 0.66 |
| **Blood Pressure** | **mmHg** | **mmHg** |  |
| Systolic BP | 104.84 | 100.80 | 0.19 |
| Diastolic BP | 63.68 | 62.69 | 0.68 |

**Table s6.** Summary Data for PLS Models Predicting DNA methylation of Immunoregulatory Genes (IL-4, IL-10, IFNγ and FoxP3) from Prior Air Pollutant Exposures. + and - indicate direct and inverse correlation, respectively. (continue on next pages)

|  | **IL-4** | | | | | |
| --- | --- | --- | --- | --- | --- | --- |
|  | CpG 3 | CpG 4 | CpG 21 | CpG 22 | CpG 23 | CpG 24 |
| Number of latent factors | 3 | 1 | 2 | 2 | 3 | 1 |
| % of variation explained by latent factors |  |  |  |  |  |  |
| For predictors (pollutants) | 37.4 | 21.2 | 35.8 | 36.3 | 43.5 | 25.1 |
| For outcome (methylation) | 20.4 | 6.9 | 33.2 | 32.4 | 50.4 | 10.9 |
| Top predictors responsible for outcome (VIP>1.3) | +:  **NO_X_** (1 day)  **NO_2_**  (1 week/month)  **PAH_456_**  (3 months)  -:  **NO_X_** (1 week) | +:  **PM_2.5_** (1 week)  **CO**  (1 week/month)  **EC** (1 day,  6 months)  -:  **NO_X_** (1 week,  3 months)  **NO_2_**  (6 to 12 month)  **PAH_456_**  (1 week) | +:  **PM_2.5_**  (1 to 3 months)  **CO**  (1 day to 3 months)  -:  **O_3_**  (1 day to 1 month) | +:  **PM_2.5_**  (1 day;  3 to 12 months)  **CO** (1 week to 6 months)  -:  **O_3_**  (1 week to 3 months) | +:  **CO**  (1 to 12 months)  -:  **O_3_**  (3 to 12 months)  **PM_2.5_**  (12 months) | +:  **PM_2.5_**  (1 week to 3 months)  **CO**  (1 day to 3months)  **EC** (6 months)  -:  **O_3_**  (1 day to 1 month) |

|  | **IL-10** | | | |
| --- | --- | --- | --- | --- |
|  | CpG 38 | CpG 39 | CpG 40 | CpG 41 |
| Number of latent factors | 1 | 1 | 1 | 1 |
| % of variation explained by latent factors |  |  |  |  |
| For predictors (air pollutants) | 25.2 | 25.9 | 25.9 | 25.8 |
| For outcome (methylation) | 9.5 | 10.1 | 11.5 | 15.1 |
| Top predictors responsible for outcome (VIP>1.3) | +:  **PM_2.5_**  (1 week to 3 months)  **CO**  (1 week to 3 months  **EC** (6 months)  -:  **O_3_**  (1 day to 1 month) | +:  **PM_2.5_**  (3 months)  **CO**  (1 day to 3 months)  -:  **O_3_**  (1 day to 1 month) **PAH_456_** (12 months) | +:  **PM_2.5_**  (1 to 3 months)  **CO**  (1 day to 3 months)  -:  **O_3_**  (1 day to 1 month)  **PAH_456_** (12 months) | +:  **PM_2.5_**  (1 week to 3 months)  **CO**  (1 day to 3 months)  -:  **O_3_**  (1 day to 1 month) |

|  | **IFNγ** | | |
| --- | --- | --- | --- |
|  | CpG 3 | CpG 4 | CpG 5 |
| Number of latent factors | 1 | 1 | 1 |
| % of variation explained by latent factors |  |  |  |
| For predictors (air pollutants) | 24.9 | 25.2 | 25.1 |
| For outcome (methylation) | 14.7 | 10.3 | 10.0 |
| Top predictors responsible for outcome (VIP>1.3) | +:  **PM_2.5_**  (1 day to 3 months)  **CO**  (1 day to 3 months)  -:  **O_3_**  (1 day to 1 month) | +:  **PM_2.5_**  (1 week to 3 months)  **CO**  (1 day to 3 months)  **EC** (6m)  -:  **O_3_**  (1 day to 1 month) | +:  **PM_2.5_**  (1 week to 3 months)  **CO**  (1 day to 3 months)  -:  **O_3_** (1 day to 1 month) |

|  | **FoxP3** | | | |
| --- | --- | --- | --- | --- |
|  | CpG 94 | | CpG 95 | |
|  | Boys | Girls | Boys | Girls |
| Number of latent factors | 1 | 1 | 1 | 1 |
| % of variation explained by latent factors |  |  |  |  |
| For predictors (pollutants) | 24.7 | 24.1 | 19.4 | 24.0 |
| For outcome (methylation) | 16.3 | 19.8 | 13.0 | 10.2 |
| Top predictors responsible for outcome (VIP>1.3) | +:  **PM_2.5_**  (1 week to 3 months)  **CO**  (1 week to 1 month)  -:  **O_3_**  (1 week to 1 month)  **NO_X_** and **NO_2_**  (12 months) | +:  **PM_2.5_**  (1 week to 6 months)  **CO**  (1 week to 1 month)  **EC** (1 week)  -:  **O_3_**  (1 day to 1 month;  12 months) | +:  **O_3_**  (6 to 12 months)  **NO_2_**  (1 month)  **EC** (1 week)  -:  **PM_2.5_** and **CO**  (6 months)  **NO_X_**  (1 month;  6 to 12 months) | +:  **PM_2.5_** (12 months)  **O_3_**  (1 day to 1 month)  **NO_2_** (1 month)  **PAH_456_**  (1 day to 1 week)  -:  **CO**  (1 day to 1 month) |

**Table S7.**  Multivariable-Adjusted Associations Between DNA Methylation of Immunoregulatory Genes (IL-4, IL-10, IFNγ and FoxP3) and Prior Air Pollutant Exposures Preselected in Partial Least Squares Analyses (continue on next pages).

| *Air pollution exposure (preselected)* | **IL-4** | | | | | |
| --- | --- | --- | --- | --- | --- | --- |
|  | CpG 3 | CpG 4 | CpG 21 | CpG 22 | CpG 23 | CpG 24 |
| PM_2.5_ |  |  |  |  |  |  |
| 1 day | n.p. | n.p. | n.p. | 0.019 (-0.10 to 0.14; P=0.76) | n.p. | n.p. |
| 1 week | n.p. | 0.061 (-0.060 to 0.18; P=0.32) | n.p. | n.p. | n.p. | 0.14 (0.020 to 0.26; P=0.022) |
| 1 month | n.p. | n.p. | 0.38 (0.26 to 0.49; P<0.0001) | n.p. | n.p. | 0.16 (0.039 to 0.28; P=0.0095) |
| 3 months | n.p. | n.p. | 0.40 (0.29 to 0.51; P<0.0001) | 0.26 (0.15 to 0.38; P<0.0001) | n.p. | 0.14 (0.019 to 0.26; P=0.023) |
| 6 months | n.p. | n.p. | n.p. | 0.34 (0.23 to 0.46; P<0.0001) | n.p. | n.p. |
| 12 months | n.p. | n.p. | n.p. | -0.29 (-0.41 to -0.17; P<0.0001) | -0.63 (-0.73 to -0.53; P<0.0001) | n.p. |
| Carbon monoxide |  |  |  |  |  |  |
| 1 day | n.p. | n.p. | 0.25 (0.13 to 0.37; P<0.0001) | n.p. | n.p. | 0.10 (-0.022 to 0.23; P=0.11) |
| 1 week | n.p. | 0.097 (-0.027 to 0.22; P=0.12) | 0.39 (0.28 to 0.51; P<0.0001) | 0.37 (0.26 to 0.49; P<0.0001) | n.p. | 0.23 (0.11 to 0.35; P=0.0002) |
| 1 month | n.p. | 0.094 (-0.033 to 0.22; P=0.15) | 0.41 (0.30 to 0.53; P<0.0001) | 0.45 (0.34 to 0.56; P<0.0001) | 0.25 (0.13 to 0.37; P<0.0001) | 0.25 (0.13 to 0.37; P<0.0001) |
| 3 months | n.p. | n.p. | 0.31 (0.19 to 0.43; P<0.0001) | 0.47 (0.36 to 0.58; P<0.0001) | 0.36 (0.25 to 0.48; P<0.0001) | 0.20 (0.081 to 0.33; P=0.0013) |
| 6 months | n.p. | n.p. | n.p. | 0.38 (0.26 to 0.49; P<0.0001) | 0.50 (0.39 to 0.61; P<0.0001) | n.p. |
| 12 months | n.p. | n.p. | n.p. | n.p. | 0.56 (0.45 to 0.67; P<0.0001) | n.p. |
| O*_3_* |  |  |  |  |  |  |
| 1 day | n.p. | n.p. | -0.34 (-0.45 to -0.22; P<0.0001) | n.p. | n.p. | -0.21 (-0.33 to -0.085; P=0.0009) |
| 1 week | n.p. | n.p. | -0.37 (-0.48 to -0.24; P<0.0001) | -0.37 (-0.48 to 0.25; P<0.0001) | n.p. | -0.21 (-0.32 to -0.087; P=0.0006) |
| 1 month | n.p. | n.p. | -0.31 (-0.43 to -0.19; P<0.0001) | -0.35 (-0.47 to -0.23; P<0.0001) | n.p. | -0.20 (-0.33 to -0.081) |
| 3 months | n.p. | n.p. | n.p. | -0.38 (-0.49 to -0.26; P<0.0001) | -0.36 (-0.48 to -0.24; P<0.0001) | n.p. |
| 6 months | n.p. | n.p. | n.p. | n.p. | -0.42 (-0.53 to -0.30; P<0.0001) | n.p. |
| 12 month | n.p. | n.p. | n.p. | n.p. | -0.065 (-0.19 to 0.056; P=0.29) | 0.055 (-0.067 to 0.18; P=0.37) |
| NOx |  |  |  |  |  |  |
| 1 day | 0.12 (-0.013 to 0.25; P=0.077) | n.p. | n.p. | n.p. | n.p. | n.p. |
| 1 week | -0.11 (-0.24 to 0.021; P=0.099) | -0.14 (-0.28 to -0.002; P=0.046) | n.p. | n.p. | n.p. | n.p. |
| 3 months | n.p. | 0.13 (-0.003 to 0.27; P=0.056)) | n.p. | n.p. | n.p. | n.p. |
| NO_2_ |  |  |  |  |  |  |
| 1 week | 0.15 (0.014 to 0.28; P=0.031) | n.p. | n.p. | n.p. | n.p. | n.p. |
| 1 month per ppb | 0.062 (-0.074 to 0.20; P=0.37) | n.p. | n.p. | n.p. | n.p. | n.p. |
| 6 months | n.p. | 0.12 (-0.02 to 0.26; P=0.10) | n.p. | n.p. | n.p. | n.p. |
| 12 months | n.p. | -0.11 (-0.25 to 0.030; P=0.12) | n.p. | n.p. | n.p. | n.p. |
| PAH_456_ |  |  |  |  |  |  |
| 1 week | n.p. | -0.11 (-0.25 to 0.028; P=0.12) | n.p. | n.p. | n.p. | n.p. |
| 3 months | 0.062 (-0.073 to 0.20;; P=0.37) | n.p. | n.p. | n.p. | n.p. | n.p. |
| EC |  |  |  |  |  |  |
| 1 day | n.p. | 0.12 (-0.022 to 0.26; P=0.10) | n.p. | n.p. | n.p. | n.p. |
| 6 months | n.p. | 0.16 (0.023 to 0.30; P=0.023) | n.p. | n.p. | n.p. | 0.25 (0.11 to 0.38; P=0.0004) |
| Values are standardized regression coefficients (for variance of dependent and independent variable equal to 1) with 95% confidence interval and P value. Effect sizes were adjusted for age, sex, asthma status, racestandardized and BMI. n.p. means not preselected in PLS analyses. | | | | | | |

| *Air pollution exposure (preselected)* | **IL-10** | | | |
| --- | --- | --- | --- | --- |
|  | CpG 38 | CpG 39 | CpG 40 | CpG 41 |
| PM_2.5_ |  |  |  |  |
| 1 week | 0.17 (0.050 to 0.29; P=0.0054) | n.p. | n.p. | 0.25 (0.14 to 0.37; P<0.0001) |
| 1 month | 0.23 (0.11 to 0.35; P=0.0001) | n.p. | 0.22 (0.10 to 0.34; 0.0003) | 0.32 (0.20 to 0.43; P<0.0001) |
| 3 months | 0.19 (0.075 to 0.31; P=0.0015) | 0.24 (0.12 to 0.35; P<0.0001) | 0.24 (0.12 to 0.35; P<0.0001) | 0.32 (0.21 to 0.43; P<0.0001) |
| Carbon monoxide |  |  |  |  |
| 1 day | n.p. | 0.18 (0.059 to 0.30; P=0.0038) | 0.20 (0.082 to 0.33; P=0.0012) | 0.25 (0.13 to 0.37; P<0.0001) |
| 1 week | 0.26 (0.14 to 0.38; P<0.0001) | 0.28 (0.16 to 0.39; P<0.0001) | 0.30 (0.19 to 0.42; P<0.0001) | 0.36 (0.25 to 0.47; P<0.0001) |
| 1 month | 0.29 (0.17 to 0.41; P<0.0001) | 0.32 (0.20 to 0.44; P<0.0001) | 0.35 (0.23 to 0.46; P<0.0001) | 0.40 (0.29 to 0.52; P<0.0001) |
| 3 months | 0.23 (0.11 to 0.35; P=0.0002) | 0.32 (0.20 to 0.44; P<0.0001) | 0.33 (0.21 to 0.45; P<0.0001) | 0.37 (0.25 to 0.48; P<0.0001) |
| *O_3_* |  |  |  |  |
| 1 day | -0.24 (-0.36 to -0.12; P<0.0001) | -0.29 (-0.41 to -0.17; P<0.0001) | -0.30 (-0.41 to -0.18; P<0.0001) | -0.37 (-0.48 to -0.25; P<0.0001) |
| 1 week | -0.27 (-0.40 to -0.15; P<0.0001) | -0.33 (-0.45 to -0.21; P<0.0001) | -0.34 (-0.46 to -0.23; P<0.0001) | -0.43 (-0.54 to -0.31; P<0.0001) |
| 1 month | -0.25 (-0.37 to -0.13; P<0.0001) | -0.33 (-0.45 to -0.21; P<0.0001) | -0.34 (-0.46 to -0.23; P<0.0001) | -0.42 (-0.53 to -0.31; P<0.0001) |
| PAH_456_ |  |  |  |  |
| 12 months | n.p. | -0.17 (-0.29 to -0.048; P=0.0065) | -0.17 (-0.29 to -0.044; P=0.0078) | n.p. |
| EC |  |  |  |  |
| 6 months | 0.20 (0.069 to 0.33) | n.p. | n.p. | n.p. |
| Values are standardized regression coefficients (for variance of dependent and independent variable equal to 1) with 95% confidence interval and P value. Effect sizes were adjusted for age, sex, asthma status, race and BMI. n.p. means not preselected in PLS analyses. | | | | |

| *Air pollution exposure (preselected)* | **IFNγ** | | |
| --- | --- | --- | --- |
|  | CpG 3 | CpG 4 | CpG 5 |
| PM_2.5_ |  |  |  |
| 1 day | 0.19 (0.067 to 0.31; P=0.0023) | n.p. | n.p. |
| 1 week | 0.21 (0.094 to 0.33; P=0.0005) | 0.099 (-0.020 to 0.22; P=0.10) | 0.15 (0.033 to 0.27; P=0.013) |
| 1 month | 0.30 (0.18 to 0.41; P<0.0001) | 0.13 (0.005 to 0.25; P=0.040) | 0.21 (0.086 to 0.33; P=0.0008) |
| 3 months | 0.26 (0.14 to 0.37; P<0.0001) | 0.10 (-0.017 to 0.22; P=0.091) | 0.16 (0.044 to 0.28; P=0.0075) |
| Carbon monoxide |  |  |  |
| 1 day | 0.27 (0.15 to 0.39; P<0.0001) | 0.13 (0.005 to 0.25; P=0.041) | 0.16 (0.039 to 0.29; P=0.010) |
| 1 week | 0.36 (0.24 to 0.47; P<0.0001) | 0.21 (0.090 to 0.33; P=0.0007) | 0.26 (0.15 to 0.38; P<0.0001) |
| 1 month | 0.40 (0.28 to 0.51; P<0.0001) | 0.25 (0.13 to 0.37; P<0.0001) | 0.30 (0.17 to 0.42; P<0.0001) |
| 3 months | 0.31 (0.19 to 0.43; P<0.0001) | 0.20 (0.079 to 0.33; P=0.0014) | 0.24 (0.11 to 0.36; P=0.0002) |
| *O_3_* |  |  |  |
| 1 day | -0.33 (-0.45 to -0.22; P<0.0001) | -0.17 (-0.28 to -0.044; P=0.0078) | -0.24 (-0.36 to -0.12; P=0.0001) |
| 1 week | -0.37 (-0.49 to -0.25; P<0.0001) | -0.21 (-0.33 to -0.083, P=0.0011)) | -0.26 (-0.38 to -0.13; P<0.0001) |
| 1 month | -0.35 (-0.46 to -0.23) | -0.20 (-0.32 to -0.076; P=0.0016) | -0.25 (-0.37 to -0.13; P<0.0001) |
| EC |  |  |  |
| 6 months | n.p. | 0.20 (0.073 to 0.33; P=0.0024) | n.p. |
| Values are standardized regression coefficients (for variance of dependent and independent variable equal to 1) with 95% confidence interval and P value. Effect sizes were adjusted for age, sex, asthma status, race and BMI. n.p. means not preselected in PLS analyses. | | | |

| *Air pollution exposure (preselected)* | **FoxP3** | | | |
| --- | --- | --- | --- | --- |
|  | CpG 94 | | CpG95 | |
|  | Boys | Girls | Boys | Girls |
| PM_2.5_ |  |  |  |  |
| 1 week | 0.21 (0.076 to 0.35; P=0.0026) | 0.14 (-0.001 to 0.28; P=0.052) | n.p. | n.p. |
| 1 month | 0.25 (0.11 to 0.39; P=0.0005) | 0.20 (0.064 to 0.34; P=0.0044) | n.p. | n.p. |
| 3 months | 0.20 (0.059 to 0.33; P=0.0054) | 0.23 (0.091 to 0.37; P=0.0015) | n.p. | n.p. |
| 6 months | n.p. | 0.20 (0.054 to 0.34; P=0.0073) | -0.18 (-0.32 to -0.039; P=0.013) | n.p. |
| 12 months | n.p. | n.p. | n.p. | 0.11 (-0.031 to 0.25 P=0.12) |
| Carbon monoxide |  |  |  |  |
| 1 day | n.p. | n.p. | n.p. | -0.19 (-0.34 to -0.043; P=0.012) |
| 1 week | 0.24 (0.093 to 0.38; P=0.0015) | 0.095 (-0.044 to 0.23; P=0.18) | n.p. | -0.24 (-0.38 to -0.099; P=0.0011) |
| 1 month | 0.24 (0.095 to 0.39; P=0.0014) | 0.12 (-0.017 to 0.278; P=0.085) | n.p. | -0.27 (-0.41 to -0.12; P=0.0004) |
| 6 months | n.p. | n.p. | -0.20 (-0.35 to -0.049; P=0.0095) | n.p. |
| *O_3_* |  |  |  |  |
| 1 day | n.p. | -0.16 (-0.30 to -0.013; P=0.033) | n.p. | 0.34 (0.19 to 0.48; P<0.0001) |
| 1 week | -0.19 (-0.33 to -0.044; P=0.011) | -0.16 (-0.31 to -0.006; P=0.041) | n.p. | 0.31 (0.15 to 0.46; P=0.0001) |
| 1 month | -0.16 (-0.30 to -0.023; P=0.023) | -0.093 (-0.25 to 0.060; P=0.23) | n.p. | 0.31 (0.15 to 0.46; P=0.0001) |
| 6 months | n.p. | n.p. | 0.13 (-0.024 to 0.28; P=0.097) | n.p. |
| 12 month | n.p. | 0.026 (-0.12 to 0.17; P=0.73) | -0.049 (-0.19 to 0.089; P=0.49) | n.p. |
| NOx |  |  |  |  |
| 1 month | n.p. | n.p. | -0.10 (-0.26 to 0.052; P=0.19) | n.p. |
| 6 months | n.p. | n.p. | -0.23 (-0.40 to -0.057; P=0.0099) | n.p. |
| 12 months | -0.20 (-0.40 to 0.001; P=0.050) | n.p. | -0.006 (-0.19 to 0.17; P=0.94) | n.p. |
| NO_2_ |  |  |  |  |
| 1 month | n.p. | n.p. | 0.22 (0.064 to 0.37; P=0.0062) | 0.17 (0.015 to 0.33; P=0.032) |
| 12 months | -0.16 (-0.34 to 0.020; P=0.080) | n.p. | n.p. | n.p. |
| PAH_456_ |  |  |  |  |
| 1 day | n.p. | n.p. | n.p. | 0.17 (0.010 to 0.32; P=0.037) |
| 1 week | n.p. | n.p. | n.p. | 0.14 (-0.004 to 0.29; P=0.057) |
| EC |  |  |  |  |
| 1 week | n.p. | -0.19 (-0.37 to -0.016) | 0.16 (0.007 to 0.32; P=0.041) | n.p. |
| Values are standardized regression coefficients (for variance of dependent and independent variable equal to 1) with 95% confidence interval and P value. Effect sizes were adjusted for age, sex, asthma status, race and BMI. n.p. means not preselected in PLS analyses. | | | | |

**Table s8.** Summary Data for PLS Models Predicting Immune Cell Typology from Prior Air Pollutant Exposures. + and - indicate direct and inverse correlation, respectively. (continue on next page)

|  | Th1 | Th2 | Th17 | Treg |
| --- | --- | --- | --- | --- |
| Number of latent factors | 2 | 1 | 1 | 1 |
| % of variation explained by latent factors |  |  |  |  |
| For predictors (pollutants) | 34.5 | 25.1 | 25.2 | 10.1 |
| For outcome (immune cell) | 33.6 | 8.5 | 12.7 | 4.3 |
| Top predictors responsible for outcome (VIP>1.3) | +:  **O_3_**  (1 week to 3 months)  -:  **PM_2.5_** (1 to 3 months)  **CO** (1 to 3 months)  **PAH_456_** (3 months) | +:  **PM_2.5_** (1 day)  **CO** (1 day to 3 months)  **NO_X_** (12 months)  -:  **O_3_** (1 week to 1 month)  **NO_2_** (3 months)  **PAH_456_** (1 day) | +:  **O_3_** (1 day to 1 month)  **PAH_456_** (1 week)  -:  **PM_2.5_** (1 week to 3 months)  **CO** (1 week to 1 month) | +:  **PM_2.5_** (12 months)  **CO** (1 day)  **O_3_** (6 months)  **PAH_456_** ( 3 months)  -:  **O_3_** (1 week)  **NO_X_** (1 day)  **NO_2_** (1 day)  **EC** (1 week) |

|  | Monocytes | B cells | CD4+ | CD8+ | CD4+/CD8+ | CD4-/CD8- |
| --- | --- | --- | --- | --- | --- | --- |
| Number of latent factors | 2 | 1 | 1 | 1 | 1 | 1 |
| % of variation explained by latent factors |  |  |  |  |  |  |
| For predictors (pollutants) | 19.5 | 11.5 | 13.8 | 15.6 | 18.3 | 9.0 |
| For outcome (immune cell) | 11.9 | 9.5 | 12.3 | 7.4 | 4.5 | 7.5 |
| Top predictors responsible for outcome | +:  **NO_2_**  (1 week/month)  **PAH_456_**  (3 months)  **EC**  (1 month)  -:  **PM_2.5_** (1 day to 1 month)  **O_3_**  (6 months) | +:  **NO_X_** (1 week; 12 months)  **PAH_456_**  (12 months)  -:  **NO_X_** (1 day)  **NO_2_** (1 month; 12 months)  **PAH_456_** (1 day to 1 week)  **EC** (6 months) | +:  **PM_2.5_** (1 week, 12 months)  **O_3_**  (3 to 12 months)  **NO_X_** (1 day)  -:  **CO**  (6 to 12 months)  **PAH_456_** (1 week) | +:  **PM_2.5_** (1 week)  **O_3_**  (3 to 6 months)  **NO_2_**  (6 months)  **PAH_456_**  (6 months)  -:  **PM_2.5_** (6 months)  **NO_X_**  (6 to 12 months)  **EC** (6 months) | +:  **PM_2.5_**  (12 months)  **O_3_** (1 day;  12 months)  **NO_X_** (1 month)  **PAH_456_**  (1 to 3 months;  12 months)  -:  **CO** (1 day) | +:  **O_3_** (1 day,  12 months)  -:  **NO_2_** (1 week, 3 months)  **PAH_456_**  (3 months)  **EC** (1 and 3 months) |

**Table S9.**  Multivariable-Adjusted Associations Between Immune Cell Typology and Prior Air Pollutant Exposures Preselected in Partial Least Squares Analyses (continue on next page).

| *Air pollution exposure (preselected)* | Th1 | Th2 | Th17 | Treg |
| --- | --- | --- | --- | --- |
| PM_2.5_ |  |  |  |  |
| 1 day | n.p. | 0.15 (0.028 to 0.27; P=0.016) | n.p. | n.p. |
| 1 week | n.p. | n.p. | -0.21 (-0.33 to -0.095; P=0.0005) | n.p. |
| 1 month | -0.34 (-0.47 to -0.21; P<0.0001) | n.p. | -0.22 (-0.35 to -0.10; P=0.0004) | n.p. |
| 3 months | -0.30 (-0.43 to -0.18; P<0.0001) | n.p. | -0.14 (-0.26 to -0.017; P=0.026) | n.p. |
| 12 months | n.p. | n.p. | n.p. | 0.0010 (-0.16 to 0.16; P=0.99) |
| Carbon monoxide |  |  |  |  |
| 1 day | n.p. | 0.16 (0.038 to 0.29; P=0.011) | n.p. | 0.10 (-0.027 to 0.23; P=0.12) |
| 1 week | n.p. | 0.16 (0.032 to 0.28; P=0.014) | -0.16 (-0.28 to -0.035; P=0.011) | n.p. |
| 1 month | -0.44 (-0.55 to -0.32; P<0.0001) | 0.18 (0.053 to 0.30; P=0.0055) | -0.16 (-0.29 to -0.043; P=0.0083) | n.p. |
| 3 months | -0.37 (-0.49 to -0.25; P<0.0001) | 0.18 (0.059 to 0.31; P=0.0040) | n.p. | n.p. |
| O*_3_* |  |  |  |  |
| 1 day | n.p. | n.p. | 0.20 (0.080 to 0.32; P=0.0013) | n.p. |
| 1 week | 0.44 (0.32 to 0.56; P<0.0001) | -0.20 (-0.32 to -0.069; P=0.0026) | 0.20 (0.075 to 0.32; P=0.0018) | -0.13 (-0.26 to -0.001; P=0.049) |
| 1 month | 0.39 (0.27 to 0.51; P<0.0001) | -0.20 (-0.32 to -0.074; P=0.0019) | 0.19 (0.068 to 0.31; P=0.0024) | n.p. |
| 3 months | 0.27 (0.14 to 0.40; P<0.0001) | n.p. | n.p. | n.p. |
| 6 months | n.p. | n.p. | n.p. | 0.058 (-0.088 to 0.20; P=0.44) |
| NOx |  |  |  |  |
| 1 day | n.p. | n.p. | n.p. | 0.04 (-0.11 to 0.19; P=0.57) |
| 12 months | n.p. | 0.22 (0.062 to 0.38; P=0.0067) | n.p. | n.p. |
| NO_2_ |  |  |  |  |
| 1 day | n.p. | n.p. | n.p. | -0.021 (-0.17 to 0.13; P=0.78) |
| 3 months | n.p. | -0.15 (-0.29 to -0.012; P=0.033) | n.p. | n.p. |
| PAH_456_ |  |  |  |  |
| 1 day | n.p. | -0.25 (-0.38 to -0.11; P=0.0004) | n.p. | n.p. |
| 1 week | n.p. | n.p. | 0.19 (0.056 to 0.33; P=0.0059) | n.p. |
| 3 months | 0.0077 (-0.067 to 0.22; P=0.29) | n.p. | n.p. | 0.11 (-0.038 to 0.26; P:0.14) |
| EC |  |  |  |  |
| 1 week | *n.p.* | *n.p.* | *n.p.* | -0.13 (-0.29 to 0.021; P=0.089) |
| Values are standardized regression coefficients (for variance of dependent and independent variable equal to 1) with 95% confidence interval and P value. Effect sizes were adjusted for age, sex, asthma status, race and BMI. n.p. means not preselected in PLS analyses. | | | | |

| *Air pollution exposure (preselected)* | Monocytes | B cells | CD4+ | CD8+ | C0D4+/CD8+ | CD4-/CD8- |
| --- | --- | --- | --- | --- | --- | --- |
| PM_2.5_ |  |  |  |  |  |  |
| 1 day | 0.23 (0.36 to 0.10; P=0.0014) | N.p. | N.p. | N.p. | N.p. | N.p. |
| 1 week | 0.12 (0.26 to -0.021; P=0.095) | N.p. | 0.12 (-0.016 to 0.27; P=0.082) | 0.079 (-0.064 to 0.22; P=0.28) | N.p. | N.p. |
| 1 month | 0.17 (0.020 to 0.32; P=0.025) | N.p. | N.p. | N.p. | N.p. | N.p. |
| 6 months | N.p. | N.p. | N.p. | -0.16 (-0.29 to -0.032; P=0.015) | N.p. | N.p. |
| 12 months | N.p. | N.p. | 0.17 (-0.031 to 0.36; P=0.098) | N.p. | 0.11 (-0.088 to 0.31; P=0.27) | N.p. |
| Carbon monoxide |  |  |  |  |  |  |
| 1 day | N.p. | N.p. | N.p. | N.p. | -0.15 (-0.30 to 0.004; P=0.057) | N.p. |
| 1 month | -0.20 (-0.35 to -0.05; P=0.0081) | N.p. | N.p. | N.p. | N.p. | N.p. |
| 6 months | N.p. | N.p. | -0.22 (-0.36 to -0.087; P=0.0015) | N.p. | N.p. | N.p. |
| 12 months | N.p. | N.p. | -0.19 (-0.34 to -0.040; P=0.013) | N.p. | N.p. | N.p. |
| *O_3_* |  |  |  |  |  |  |
| 1 day | N.p. | N.p. | N.p. | N.p. | -0.059 (-0.21 to 0.091; P=0.44) | 0.088 (-0.062 to 0.24; P=0.25) |
| 3 months | N.p. | N.p. | 0.14 (-0.0057 to 0.29; P=0.059) | 0.14 (-0.005 to 0.29; P=0.058) | N.p. | N.p. |
| 6 months | -0.12 (-0.28 to 0.040; P=0.14) | N.p. | 0.26 (0.11 to 0.42; P=0.0008) | 0.15 (-0.007 to 0.31; P=0.061) | N.p. | N.p. |
| 12 month | N.p. | N.p. | 0.14 (-0.002 to 0.28; P=0.054) | N.p. | 0.0004 (-0.15 to 0.15; P=0.99) | 0.002 (-0.14 to 0.15; P=0.97) |
| NOx |  |  |  |  |  |  |
| 1 day | N.p. | -0.19 (-0.33 to -0.047; P=0.0093) | 0.16 (0.015 to 0.31; P=0.031) | N.p. | N.p. | N.p. |
| 1 week | N.p. | 0.076 (-0.062 to 0.21; P=0.28) | N.p. | N.p. | N.p. | N.p. |
| 1 month | N.p. | N.p. | N.p. | N.p. | 0.11 (-0.037 to 0.26; P=0.14) | N.p. |
| 6 months | N.p. | N.p. | N.p. | -0.20 (-0.35 to -0.049; P=0.0096) | N.p. | N.p. |
| 12 months | N.p. | 0.13 (-0.042 to 0.31; P=0.14) | N.p. | 0.15 (-0.024 to 0.33; P=0.090) | N.p. | N.p. |
| NO_2_ |  |  |  |  |  |  |
| 1 week | 0.11 (-0.039 to 0.25; P=0.15) | N.p. | N.p. | N.p. | N.p. | -0.081 (-0.23 to 0.063; P=0.27) |
| 1 month | 0.011 (-0.14 to 0.16; P=0.88) | -0.082 (-0.23 to 0.061; P=0.26) | N.p. | N.p. | N.p. | N.p. |
| 3 months | N.p. | N.p. | N.p. | N.p. | N.p. | -0.11 (-0.26 to 0.033; P=0.13) |
| 6 months | N.p. | N.p. | N.p. | 0.10 (-0.048 to 0.25; P=0.18) | N.p. | N.p. |
| 12 months | N.p. | -0.058 (-0.21 to 0.091; P=0.44) | N.p. | N.p. | N.p. | N.p. |
| PAH_456_ |  |  |  |  |  |  |
| 1 day | N.p. | -0.12 (-0.26 to 0.024; P=0.10) | N.p. | N.p. | N.p. | N.p. |
| 1 week | N.p. | -0.079 (-0.22 to 0.060; P=0.26) | -0.19 (-0.33 to -0.05; P=0.0080) | N.p. | N.p. | N.p. |
| 1 month | N.p. | N.p. | N.p. | N.p. | 0.065 (-0.083 to 0.21; P=0.39) | N.p. |
| 3 months | 0.019 (-0.13 to 0.17; P=0.81) | N.p. | N.p. | N.p. | 0.055 (-0.095 to 0.21; P=0.47) | -0.11 (-0.25 to 0.041; P=0.16) |
| 6 months | N.p. | N.p. | N.p. | 0.16 (0.009 to 0.31; P=0.038) | N.p. | N.p. |
| 12 months | N.p. | 0.13 (-0.023 to 0.28; P=0.096) | N.p. | N.p. | -0.077 (-0.23 to 0.077; P=0.32) | N.p. |
| EC |  |  |  |  |  |  |
| 1 month | 0.13 (-0.013 to 0.28; P=0.074) | N.p. | N.p. | N.p. | N.p. | -0.14 (-0.29 to 0.000433, P=0.051) |
| 3 months | N.p. | N.p. | N.p. | N.p. | N.p. | -0.10 (-0.25 to 0.042; P=0.16) |
| 6 months | N.p. | -0.15 (-0.24 to 0.038; P=0.15) | N.p. | -0.14 (-0.29 to 0.002; P=0.053) | N.p. | N.p. |
| Values are standardized regression coefficients (for variance of dependent and independent variable equal to 1) with 95% confidence interval and P value. Effect sizes were adjusted for age, sex, asthma status, race and BMI. n.p. means not preselected in PLS analyses. | | | | | | |

**Table S10.**  Multivariable-Adjusted Associations Between Blood Pressure and Prior Air Pollutant Exposures Preselected in Partial Least Squares Analyses.

| *Air pollution exposure (preselected)* | Systolic BP | Diastolic BP | Pulse pressure |
| --- | --- | --- | --- |
| PM_2.5_ |  |  |  |
| 3 months | n.p. | -0.033 (-0.14 to 0.071; P=0.53) | n.p. |
| 6 months | n.p. | -0.13 (-0.23 to -0.020; P=0.020) | -0.032 (-0.15 to 0.083; P=0.59) |
| Carbon monoxide |  |  |  |
| 3 months | n.p. | -0.081 (-0.19 to 0.027; P=0.14) | 0.025 (-0.091 to 0.14; P=0.67) |
| 6 months | n.p. | -0.17 (-0.27 to -0.059; P=0.0024) | n.p. |
| 12 months | n.p. | n.p. | 0.023 (-0.10 to 0.14; P=0.70) |
| *O_3_* |  |  |  |
| 1 month | n.p. | n.p. | -0.028 (-0.14 to 0.086; P=0.63) |
| 12 month | -0.036 (-0.14 to 0.067; P=0.49) | *n.p.* | -0.13 (-0.25 to -0.017; P=0.025) |
| NOx |  |  |  |
| 1 week | *n.p.* | 0.12 (-0.008 to 0.24; P=0.068) | *n.p.* |
| 1 month | *n.p.* | -0.020 (-0.15 to 0.11; P=0.76) | *n.p.* |
| 3 months | 0.17 (0.046 to 0.29; P=0.0073) | *n.p.* | 0.15 (0.010 to 0.28; P=0.035) |
| 12 months | *n.p.* | *n.p.* | -0.16 (-0.30 to -0.025; P=0.021) |
| NO_2_ |  |  |  |
| 1 day | 0.13 (0.004 to 0.25; P=0.043) | *n.p.* | 0.14 (0.009 to 0.28; P=0.036) |
| PAH_456_ |  |  |  |
| 1 week | 0.076 (-0.048 to 0.20; P=0.23) | *n.p.* | *n.p.* |
| 1 month | *n.p.* | -0.099 (-0.23 to 0.028; P=0.13) | *n.p.* |
| 6 months | 0.13 (0.008 to 0.26; P=0.037) | 0.17 (0.045 to 0.30; P=0.0081) | *n.p.* |
| EC |  |  |  |
| 1 month | *n.p.* | -0.067 (-0.19 to 0.057; P=0.29) | *n.p.* |
| Values are standardized regression coefficients (for variance of dependent and independent variable equal to 1) with 95% confidence interval and P value. Effect sizes were adjusted for age, sex, asthma status, race and BMI. n.p. means not preselected in PLS analyses. | | | |

**Figure s1:** Longitudinal measurement of air pollutants including PM_2.5_, O_3_, PAH_456_, NO_2_, Elemental Carbon, NOx, and CO in five different stations at Fresno, California.


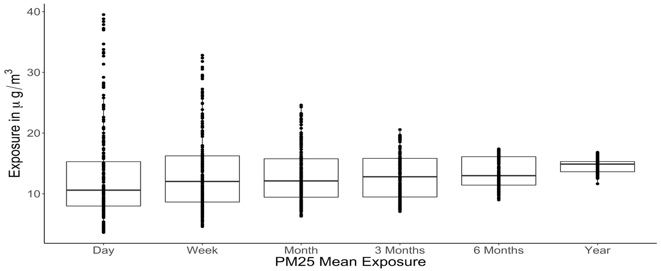


Exposure in ppb

Exposure in υg/m^3^

Day Week Month 3 Mon 6 Mon Year

Mean **PM_2.5_** Exposure


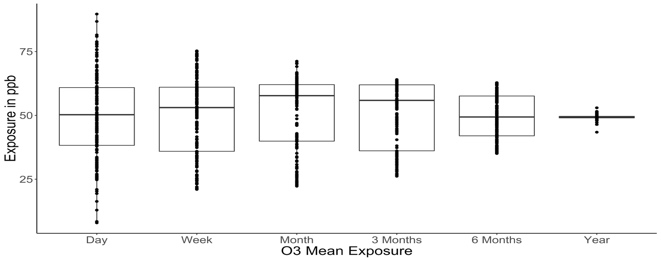


Exposure in ppb

Day Week Month 3 Mon 6 Mon Year

Mean **O_3_** Exposure


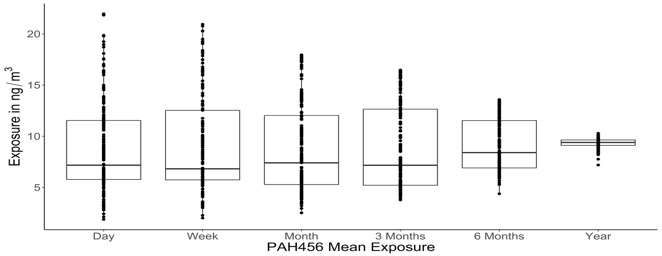


Exposure in ng/m^3^

Day Week Month 3 Mon 6 Mon Year

Mean **PAH_456_** Exposure


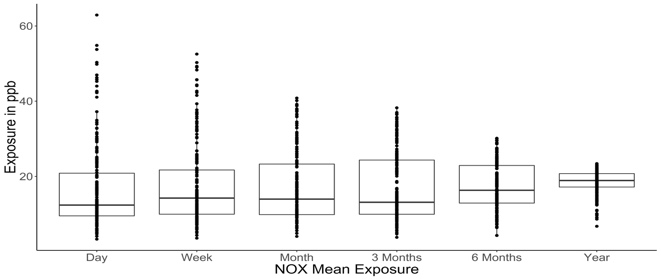


Day Week Month 3 Mon 6 Mon Year

Mean **NO_2_** Exposure


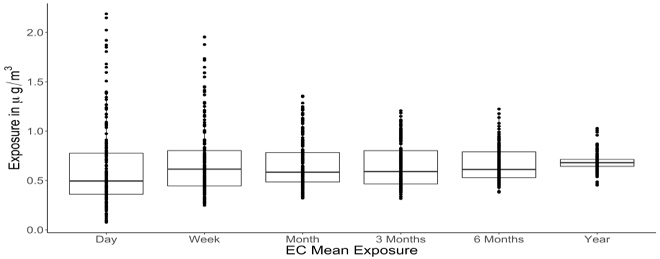


Day Week Month 3 Mon 6 Mon Year

Mean **EC** Exposure

Exposure in υg/m^3^


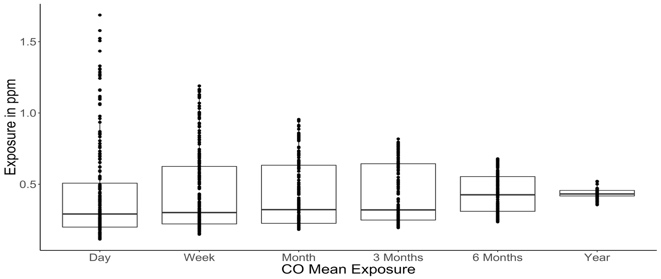


Day Week Month 3 Mon 6 Mon Year

Mean **CO** Exposure

Exposure in ppm


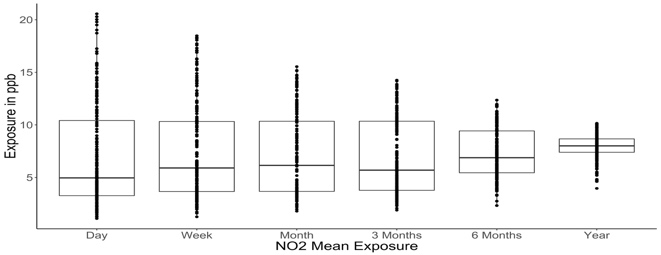
**Figure s2:** Schematic of CyTOF workflow. PBMCs, peripheral blood mononuclear cells

Exposure in ppb

Day Week Month 3 Mon 6 Mon Year

Mean **NOx** Exposure


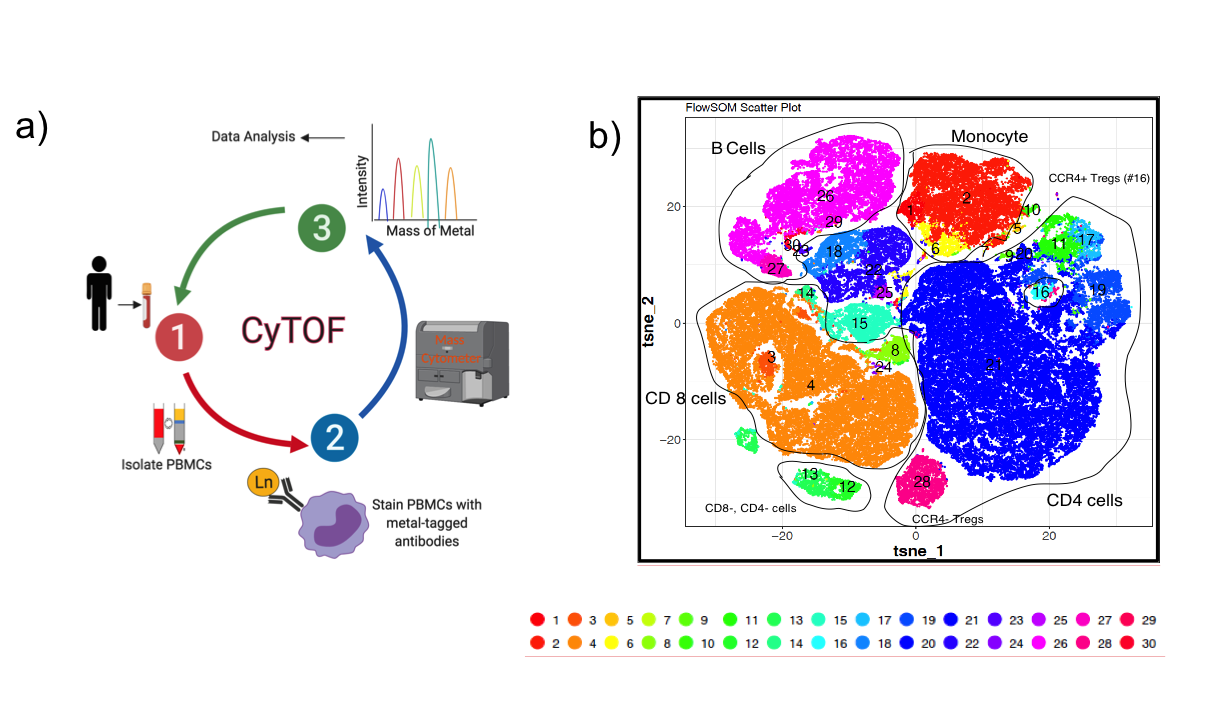


**Figure s3**: Heat map of FlowSOM Cluster Analysis shows the median intensities of 12 surface markers across different immune cell populations after the meta-clustering of PBMCs from minority children exposed to ambient air pollutants. Blue color represents the lowest and red color represents the highest intensity/expression of each marker examined (n = 191).

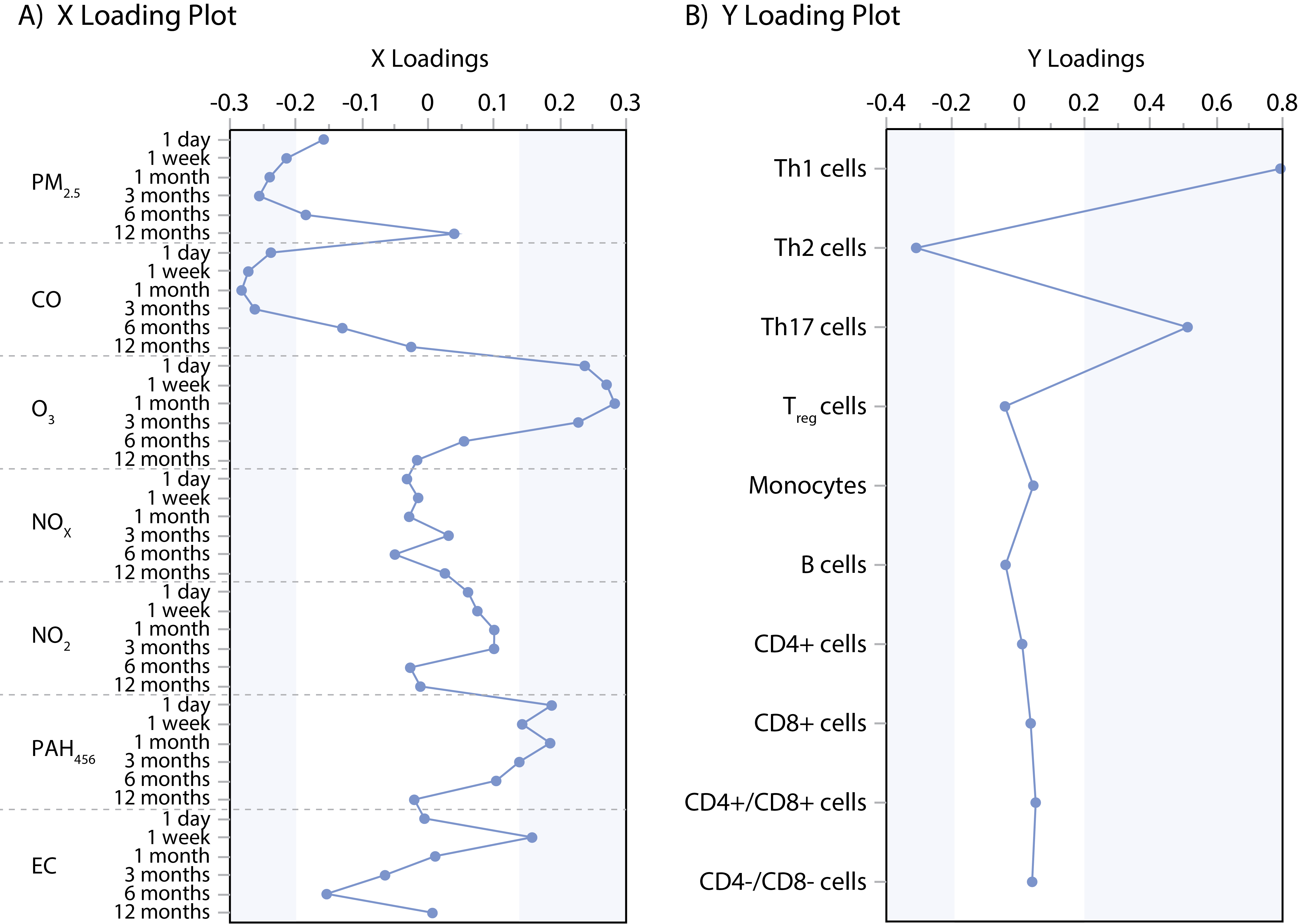
**Figure S4. Loading Plots from the Multivariate Partial Least Squares (PLS) Modeling to Identify Key Air Pollutant Exposure for Prediction of Key Immune Cell Types.** Higher loadings (positive and negative) indicates higher weight of the variable on the latent factors constructed during PLS and thus highlights a higher influence of the variable on the overall model. Variables in the shaded area were considered as key predictors (A) and outcome (B).
